# Dissection of QTLs conferring drought tolerance in *B. carinata* derived *B. juncea* introgression lines

**DOI:** 10.1186/s12870-023-04614-z

**Published:** 2023-12-21

**Authors:** Omkar Maharudra Limbalkar, Prashant Vasisth, Guman Singh, Priyanka Jain, Mohit Sharma, Rajendra Singh, Gokulan Dhanasekaran, Manish Kumar, Mohan Lal Meena, Mir Asif Iquebal, Sarika Jaiswal, Mahesh Rao, Anshul Watts, Ramcharan Bhattacharya, Kunwar Harendra Singh, Dinesh Kumar, Naveen Singh

**Affiliations:** 1 Division of Genetics, Indian Council of Agricultural Research (ICAR)-Indian Agricultural Research Institute, New Delhi, India; 2 Present Address: ICAR-Indian Institute of Agricultural Biotechnology, Ranchi, Jharkhand India; 3 ICAR-Directorate of Rapeseed-Mustard Research, Sewar, Bharatpur, Rajasthan India; 4 Division of Agricultural Bioinformatics, ICAR-Indian Agricultural Statistics Research Institute, New Delhi, India; 5 Present Address: AIMMSCR, Amity University Uttar Pradesh, Sector 125, Noida, Uttar Pradesh 201313 India; 6 Present Address: College of Agriculture, Navgaon, Alwar, Sri Karan Narendra Agriculture University, Jobner, Rajasthan India; 7 ICAR-National Institute for Plant Biotechnology, New Delhi, India; 8 Present Address: ICAR, Indian Institute of Soybean Research, Indore, Madhya Pradesh India

**Keywords:** Indian mustard, *Brassica carinata*, Interspecific hybridization, Water use efficiency, Drought tolerance indices, QTL hotspot

## Abstract

**Background:**

Drought is one of the important abiotic stresses that can significantly reduce crop yields. In India, about 24% of *Brassica juncea* (Indian mustard) cultivation is taken up under rainfed conditions, leading to low yields due to moisture deficit stress. Hence, there is an urgent need to improve the productivity of mustard under drought conditions. In the present study, a set of 87 *B. carinata*-derived *B. juncea* introgression lines (ILs) was developed with the goal of creating drought-tolerant genotypes.

**Method:**

The experiment followed the augmented randomized complete block design with four blocks and three checks. ILs were evaluated for seed yield and its contributing traits under both rainfed and irrigated conditions in three different environments created by manipulating locations and years. To identify novel genes and alleles imparting drought tolerance, Quantitative Trait Loci (QTL) analysis was carried out. Genotyping-by-Sequencing (GBS) approach was used to construct the linkage map.

**Results:**

The linkage map consisted of 5,165 SNP markers distributed across 18 chromosomes and spanning a distance of 1,671.87 cM. On average, there was a 3.09 cM gap between adjoining markers. A total of 29 additive QTLs were identified for drought tolerance; among these, 17 (58.6% of total QTLs detected) were contributed by *B. carinata* (BC 4), suggesting a greater contribution of *B. carinata* towards improving drought tolerance in the ILs. Out of 17 QTLs, 11 (64.7%) were located on the B genome, indicating more introgression segments on the B genome of *B. juncea*. Eight QTL hotspots, containing two or more QTLs, governing seed yield contributing traits, water use efficiency, and drought tolerance under moisture deficit stress conditions were identified. Seventeen candidate genes related to biotic and abiotic stresses, viz., *SOS2*, *SOS2 like*, *NPR1*, *FAE1-KCS*, *HOT5*, *DNAJA1*, *NIA1*, *BRI1*, *RF21*, y*cf2*, *WRKY33*, *PAL*, *SAMS2*, *orf147*, *MAPK3*, *WRR1* and *SUS*, were reported in the genomic regions of identified QTLs.

**Conclusions:**

The significance of *B. carinata* in improving drought tolerance and WUE by introducing genomic segments in Indian mustard is well demonstrated. The findings also provide valuable insights into the genetic basis of drought tolerance in mustard and pave the way for the development of drought-tolerant varieties.

**Supplementary Information:**

The online version contains supplementary material available at 10.1186/s12870-023-04614-z.

## Background

Global warming and climate change present a severe threat to crop production, including the occurrence of various biotic and abiotic stresses [1, 2]. Drought, among the abiotic stresses, significantly impacts the growth and productivity of crop plants [3, 4]. Over the last 6–7 decades, India has experienced a consistent decline in summer monsoon rainfall, leading to an increased risk of droughts [5]. Between 1951 and 2016, droughts became more frequent and widespread, particularly in central India, the southwest coast, the southern peninsula, and the north-eastern parts of the country, which experienced an average of two droughts per decade [5]. Furthermore, the drought-affected area has expanded by 1.3% per decade during this period [5]. Rapeseed-mustard, an important group of edible oilseed crops, is cultivated worldwide and occupies 41.64 million hectares (Mha) with a production of approximately 87.30 million metric tons (MMT). It contributes 13.91% to global oilseed production (627.44 MMT). In India, Indian mustard (*B. juncea*) dominates the rapeseed-mustard group, covering over 90% of the total acreage (9.00 Mha) and accounting for 28.45% of the country's oilseed production (40.42 MMT) in 2022–23 [6].

Higher production of Indian mustard is needed to meet the edible oil demand of the ever-growing population, which can be well achieved by improving productivity and reducing the yield losses caused by different biotic and abiotic stresses. Efforts have been made in the past to improve seed and oil yields to achieve self-sufficiency, but a huge quantity of edible oil is still imported annually. According to the Indian Vegetable Oil Producers’ Association, India imported 14.38 million metric tons (MMT) of oil, costing over 18 billion dollars accounting for more than 70% of the country's total edible oil demand in the year 2022–23 (Source: https://www.indiastatagri.com/). The rise in per capita oil consumption is driven by factors like population growth, increasing income and changing dietary preferences, and is expected to further increase [7]. To meet the edible oil demand of growing population, it is estimated that approximately 16.4–20.5 MMT of rapeseed-mustard needs to be produced [8, 9], while the current production stands at only 11.5 MMT [6].

To achieve self-sufficiency in edible oil, the productivity of Indian mustard needs to be urgently improved. However, the narrow genetic base of this species poses a significant constraint to its improvement [10]. The crop's susceptibility to various pests, diseases, and environmental stresses contributes to inconsistent production patterns. Approximately 24% of Indian mustard cultivation is taken up in rainfed areas, resulting in substantial yield losses due to moisture deficit stress [11]. Most commercially released *B. juncea* varieties are sensitive to drought, leading to critical seed yield losses. Although a few drought-tolerant varieties have been developed for drought-prone regions, their poor yield potential limits adoption. Therefore, the search for new sources imparting drought tolerance is urgently needed to reduce yield losses, particularly in the drought-prone areas of the eastern and western parts of the country [12].

*Brassica* species and their close relatives possess beneficial traits such as tolerance to cold, salinity and drought, which could be incorporated into present-day cultivars [13–18]. Wide hybridization has been considered a novel approach for the successful transfer of desirable traits and generating selectable genetic variability in cultivated species [19–21]. Ethiopian mustard, *B. carinata* (BBCC; 2n = 4x = 34), possesses resistance/tolerance to various abiotic and biotic stresses, including drought, heat, aphid, white rust, *Sclerotinia* rot, *Alternaria* black spot, powdery mildew and blackleg [22, 23]. Exploiting *B. carinata* as a donor for drought tolerance can enhance the performance and stability of Indian mustard cultivars under diverse arid environments [23–25].

Drought tolerance is a complex, polygenic trait reported in several crop species, including mustard [18]. To improve drought tolerance, approaches like introgressions from wild relatives, quantitative trait loci (QTL) mapping and marker-assisted backcross breeding (MABB) are being employed [26]. QTL mapping requires a large number of molecular markers with genome-wide coverage for high resolution. Simple sequence repeats (SSRs) have been extensively used for QTL mapping [27], but their limited number and uneven distribution in the genome hinder resolution. This has been greatly achieved by the advent of next-generation sequencing (NGS) techniques.

Next generation sequencing platforms offer a cost-effective solution for genotyping by sequencing single nucleotide polymorphic (SNP) sites, offering thousands to millions of molecular markers. Whole genome resequencing is an effective method when a reference genome is available, but it can be costly for crop species with large genomes. Therefore, genotyping-by-sequencing (GBS), a reduced representation genotyping approach based on restriction site-associated DNA sequencing (RAD-seq), has emerged as a novel and alternative method [28]. It has been successfully employed for high-resolution linkage mapping, particularly in introgression lines derived from related species of *Brassicas* [28–30]. However, very few studies reported QTLs associated with drought tolerance in rapeseed-mustard [18, 31];) and the mapping of QTLs for drought tolerance or improved water use efficiency in *B. carinata* or its derived lines remain largely unexplored.

With a view to improve the ability of *B. juncea* to withstand moisture deficit stress conditions, efforts were made at the Indian Council of Agricultural Research-Indian Agricultural Research Institute (ICAR-IARI), New Delhi to broaden the genetic base of *B. juncea* through the development of introgression lines (ILs) carrying genomic segments from *B. carinata*. The present study also aimed to identify QTLs/genes responsible for imparting drought tolerance and/or improved water use efficiency (WUE). To the best of our knowledge, this is the first instance where ILs possessing useful genomic segment(s) from *B. carinata* were developed and deployed for genetic studies conducted under moisture deficit stress conditions. This diverse set of ILs, evaluated under moisture deficit stress conditions in different environments, was used to map the QTL(s) and decipher the underlying candidate genes associated with WUE, drought tolerant indices and yield contributing traits. This study will lay the foundation for future investigations into molecular mechanisms underlying drought tolerance/WUE in mustard.

## Materials and methods

### Development and cytological investigation on *B. carinata* derived *B. juncea* introgression lines

**Fig. 1:**
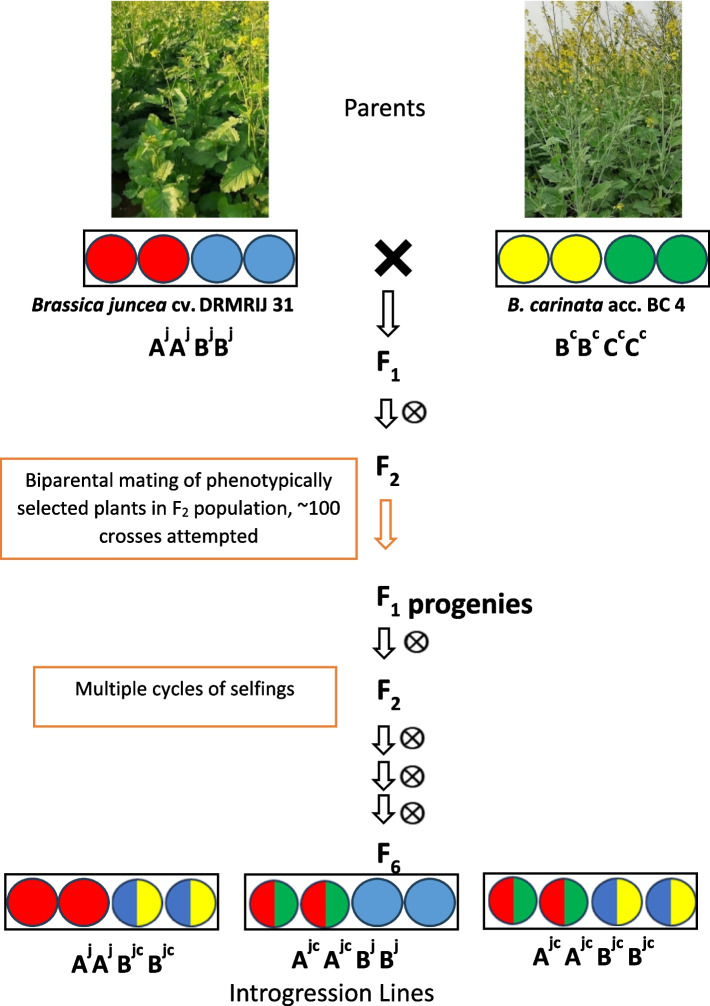
Scheme for development of introgression lines in genetic background of DRMRIJ 31 (^j^ and ^c^ represent genome from *B. juncea* and *B. carinata* parent, respectively)

A set of 87 *B. juncea* introgression lines, in F_6_ generation, carrying the genomic segments of *B. carinata* was developed. These lines were derived from the cross between *B. juncea* cv. DRMRIJ 31 and *B. carinata* acc. BC 4, and following biparental mating among phenotypically selected plants within F_2_ population (Fig. 1). Phenotypic selection of desirable segregants having a close resemblance to *B. juncea* parent in the subsequent filial generations led to the development of *B. carinata* derived *B. juncea* introgression lines (ILs). These ILs were identified as homozygous and cytologically stable, as reported in our earlier study on the expression of heterosis by them [32]. Further, molecular analysis on these ILs has confirmed the presence of introgression segments from *B. carinata* [33].

### Phenotypic evaluation of ILs under moisture deficit stress conditions

A set of ILs along with their parents were evaluated at ICAR-IARI, New Delhi (L-1) and ICAR-Directorate of Rapeseed and Mustard Research (ICAR-DRMR), Bharatpur, Rajasthan (L-2). During 2018–19 crop season, the experiment was conducted at L1 location under rainfed (RE1) and irrigated (IE1) conditions, whereas during 2020–21 crop season it was done at both L1 and L2 locations under rainfed (RE2 and RE3) and irrigated (IE2 and IE3) conditions, thus consisting of a total of six environments. An augmented Randomised Complete Block Design (RCBD) with four blocks and three checks (Pusa Agrani, DRMRIJ 31 and Pusa Mustard 30) was used for the morphological characterization of ILs differing in metric traits and response to moisture deficit stress. Checks were replicated in each block under both rainfed and irrigated conditions. Each IL was raised in a paired row plot of four meters in length. Within each plot, row-row and plant-plant spacing were kept at 30 cm and 15 cm, respectively, and plots were separated by 60 cm. No irrigation was applied in the rainfed plots, while two irrigations of 50 mm were applied in the irrigated plots at 45 and 90 days after sowing (DAS). All recommended agronomic practices were followed for raising the crop.

Data were recorded on 15 quantitative traits, viz., plant height (cm), primary branches/plant, secondary branches/plant, main shoot length (cm), total siliquae on main shoot, siliqua length (cm), seeds/siliqua, seed yield/plant (g), biological yield/plant (g), seed yield/plot (g), harvest index, oil content (%), 1,000-seed weight (g), days to 50% flowering, and days to maturity. The data were recorded on five randomly selected competitive plants from each plot in each replication, except for seed yield per plot (g), days to 50% flowering and days to maturity, where observations were recorded on a plot basis. The biological yield/plant (g) was recorded at maturity after completely drying the harvested plants.

### Statistical analysis

An analysis of variance for augmented randomized complete block design (Augmented-RCBD) was performed for all the studied traits following the statistical model,

$${Y}_{ijk}= \mu +G +C + {E}_{i} +G \times {E}_{i} +C \times {E}_{i} + {B}_{k}\left({E}_{i}\right) + {e}_{ilk}$$

where, *Y*_*ijk*_ is the mean values of a trait in *i*^th^ environment of *j*^th^ genotype in* k*^th^ block; *μ* is the population mean; G, C and E_*i*_ is genotype, check and *i*^th^ environment, respectively; G × E_*i*_ and C × E*i* is the effect of the genotype by environment interaction and check by environment interaction, respectively; B_*k*_ (E_*i*_) is the effect of the *k*^th^ block within *i*^th^ environment; and *e*_*ilk*_ is the residual error.

Best linear unbiased predictors (BLUP) values for all studied traits were estimated to perform combined analysis under both rainfed and irrigated conditions across the environments using Augmented Complete Block Design in R (ACBD-R) software [34, 35]. The descriptive statistics such as mean, range and broad-sense heritability ((h^2^); [36]) were estimated using Plant Breeding Tools software [37].

## Assessment of drought tolerance

Adjusted mean values of all the studied traits under both rainfed and irrigated conditions were used for the estimation of drought susceptibility index (DSI), drought tolerance index (DTI), tolerance index (TOL) and mean relative performance (MRP) using the following formulae:

- Drought Susceptibility Index (DSI) = (1–Y_s_ / Y_ns_) / D; [38]
- Drought tolerance index (DTI) = (Y_s_ × Y_ns_) */ (*Y_nsm_).^2^; [39]
- Tolerance index (TOL) = Y_ns_–Y_s_; [40]
- Mean relative performance: MRP = [Y_s_/Y_sm_ + Y_ns_/Y_nsm_]

Where, D is stress intensity and calculated as, D = 1– (Y_sm_ / Y_nsm_); Y_s_ = seed yield of genotype under moisture deficit stress conditions (drought stress); Y_ns_ = seed yield of genotype under irrigated or non-stress conditions; Y_sm_ = mean seed yield of all genotypes under moisture deficit stress conditions; Y_nsm_ = mean seed yield of all genotypes under irrigated or non-stress conditions.

Water-use efficiency (WUE) for ILs was estimated from the following formulae:

$$\mathrm{WUE} \left[\mathrm{kg }{\mathrm{ha}}^{-1}{\mathrm{mm}}^{-1}\right]= \frac{\mathrm{Seed Yield }(\mathrm{kg}/\mathrm{ha})}{\mathrm{Water received from irrigation and rainfall }(\mathrm{mm})}$$

$$\mathrm{WUE} \left[\mathrm{kg }{\mathrm{m}}^{-3}\right]=0.1 \times \mathrm{WUE }\left[\mathrm{kg }{\mathrm{ha}}^{-1}{\mathrm{mm}}^{-1}\right]$$

**Fig. 2:**
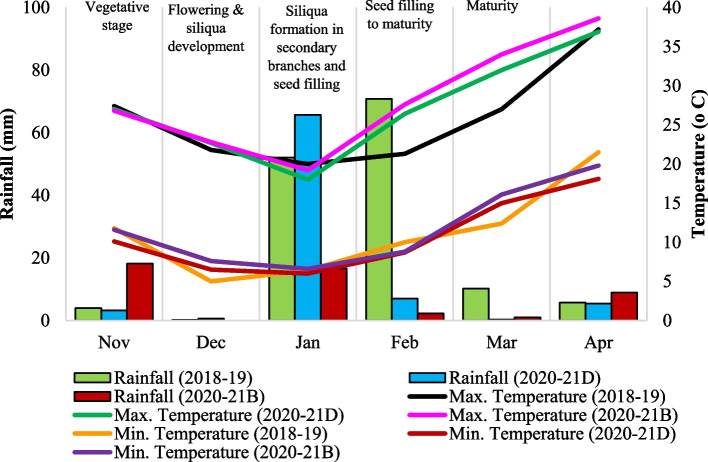
The maximum, minimum temperatures and rainfall recorded from the experimental site during crop growth seasons of 2018–19 and 2020–21

The minimum and maximum temperatures (°C) and rainfall (mm) were recorded during the crop growth seasons during 2018–19 and 2020–21 by the meteorological observatories located within 500 m distance from the trial sites at ICAR-IARI, New Delhi and ICAR-DRMR, Bharatpur (Fig. 2). The effective rainfall during crop season from November to March was calculated for respective season using Cropwat software (Version 8.0) by USDA SCS method. The water use efficiency in ILs was then estimated using effective rainfall observed.

### Genotyping by sequencing

A total of 87 introgression lines (ILs) were used for genotyping by sequencing [GBS; [41]]. The genomic DNA was extracted from each genotype following the modified Cetyl Trimethyl Ammonium Bromide (CTAB) method suggested by Saghai-Maroof et al. [42]. Genomic services were outsourced from NxGenBio Life Sciences Private Limited. An optimized GBS library was prepared using high-quality DNA following the protocol given by Elshire et al*.* [41], and raw sequence data were generated using Illumina True Seq sequencing. Sequence reads were processed and aligned to the reference genome of *B. juncea* cv. Varuna UDSC Var 1.1 [43] using bwa version 0.7.17 [44]. Polymorphic SNPs were identified using the TASSEL-GBS pipeline [45], which further filtered for minor allele frequency of > 0.05 as well as missing genotypic frequency of < 0.01. The linkage map was constructed using polymorphic non-redundant binned SNPs with the least missing data using IciMapping 4.2 tool ([http://www.isbreeding.net; [46]).

### Mapping of QTLs and candidate gene analysis

Agro-morphological traits recorded on ILs across the environments and molecular data were used to map Quantitative Trait Loci (QTL) in different environments using BIP and MET function in IciMapping 4.2 software [46]. To increase the authenticity and reliability of the detected QTLs, Logarithm of the Odds (LOD) was kept at ≥ 3. QTLs were named as per the previously described method [47, 48]. The name of the identified QTL begins with the initial "*q*" followed by the short form of the trait and the linkage group (A1 to A10 and B1 to B8). When any linkage group contains more than one QTL, the detected QTLs are numbered in order of their physical locations. Candidate genes within the region of identified QTLs were predicted following the method suggested by Wang et al. [49]. The identification of candidate genes was based on the annotation of the *Arabidopsis thaliana* genome and the physical positions of SNPs in the *B. juncea* reference genome [43].

## Results

### Phenotypic evaluation of introgression lines

**Fig. 3:**
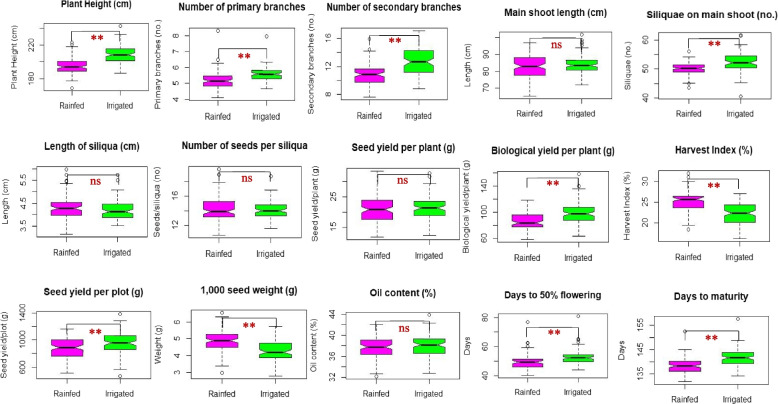
Notched box plots for agro-morphological traits in introgression lines (ILs) of DRMRIJ 31 genetic background under rainfed and irrigated conditions. *Box edges represent upper and lower quartile, with median value shown as a bold line in the middle of the box. Whiskers represent 1.5 times the quartile of the data. Individuals falling outside the range of the whiskers shown as open dots. Boxplot obtained between two water regimes for each trait was compared using Wilcoxon statistic, and corresponding level of significance was shown by *P* values in codes (**p ≤ 0.01, *p ≤ 0.05, ns, non-significant)

Wide range of phenotypic variations were observed for all the 15 agro-morphological traits in the ILs evaluated under both rainfed and irrigated conditions. All the traits were normally distributed (Fig. 3), indicating the suitability of ILs for QTL mapping. The notched boxplots obtained between rainfed and irrigated conditions for each trait were compared following the Wilcoxon test, with the corresponding level of significance depicted through *P* values (Fig. 3). Pooled analysis over the environments indicated a significant mean difference between rainfed and irrigated conditions for studied traits, viz., plant height (cm), primary branches/plant, secondary branches/plant, total siliquae on main shoot, biological yield per plant (g), harvest index, seed yield per plot (g), 1,000-seed weight (g), days to 50% flowering and days to maturity. The mean values of harvest index and 1,000 seed weight (g) were found to be significantly higher under rainfed than in irrigated conditions (Fig. 3).

The data recorded on all the traits under three different environments were pooled, and analysis revealed a significant effect of genotypes (*P* ≤ 0.01) under both rainfed and irrigated conditions (Table 1). This indicates the presence of sufficient genetic variability in the ILs for all the studied traits across environments. Significant G × E interactions were also observed for all the traits studied (Table 1). A wide range of variation, along with transgressive segregants, was recovered for almost all the traits recorded under rainfed and irrigated conditions (Table 1). The broad-sense heritability across the environments ranged from 0.19 to 0.95 and from 0.28 to 0.91 under rainfed and irrigated conditions, respectively. Under rainfed conditions, all the traits exhibited high heritability except for secondary branches per plant, seed yield per plant and biological yield per plant, which exhibited low to moderate heritability (Table 1).

**Table 1:** Analysis of variance, mean, range and broad sense heritability of introgression lines (ILs) in DRMRIJ 31 genetic background for yield contributing traits under rainfed (RF) and irrigated (IR) conditions across the environments

Traits		Sources of variations							Mean			Range in ILs	h^2^	CV (%)
		Genotype		Environment		Genotype × Environment		Error variance	DRMRIJ-31	BC 4	ILs			
		F value	*P–* value	χ^2^	*P–* value	χ^2^	*P–* value							
PH	RF	3.24**	0.000	16.64**	0.000	3.95**	0.001	161.40	192.16	216.10	195.02	168.8–222.8	0.81	9.20
	IR	4.57**	0.000	13.23**	0.000	4.65**	0.004	139.20	209.26	228.00	208.75	186.4–242.6	0.89	10.60
PB/pt	RF	2.73**	0.000	19.16**	0.000	51.36**	0.000	0.11	4.68	8.31	5.17	4.1–6.5	0.68	9.10
	IR	1.58**	0.008	9.32**	0.002	6.34*	0.012	0.42	4.99	7.97	5.56	4.7–6.4	0.48	14.90
SB/pt	RF	2.15**	0.002	2.26 ns	0.132	55.17**	0.000	0.98	9.79	15.92	10.77	7.6–14.4	0.20	6.40
	IR	1.87**	0.000	2.06 ns	0.151	43.33**	0.000	1.17	12.52	16.44	12.67	8.8–17.1	0.49	11.30
MSL	RF	2.92**	0.000	11.77**	0.000	7.28**	0.007	35.60	83.06	76.52	82.79	65.3–97.0	0.76	9.40
	IR	2.05**	0.000	9.59**	0.002	23.30**	0.000	39.65	84.68	95.38	83.93	72.1–101.9	0.60	11.30
SoMS	RF	1.92**	0.000	2.44 ns	0.118	3.76**	0.052	26.60	49.91	53.88	50.15	43.6–56.1	0.64	6.00
	IR	1.63**	0.003	0.93 ns	0.335	24.87**	0.000	11.70	53.29	58.26	52.44	40.6–61.7	0.45	5.90
Siliqua length	RF	11.03**	0.000	16.12**	0.000	30.14**	0.000	0.03	4.23	4.00	4.31	3.1–6.0	0.95	6.70
	IR	6.02**	0.000	9.09**	0.002	14.55**	0.000	0.08	4.18	3.94	4.19	3.5–5.7	0.90	7.20
Seeds/ Siliqua	RF	5.92**	0.000	10.20**	0.001	17.38**	0.000	0.52	15.32	15.43	14.16	10.7–19.6	0.87	10.00
	IR	4.02**	0.000	6.54*	0.011	4.41*	0.035	1.19	14.90	14.52	14.03	11.6–18.6	0.86	4.70
SY/pt	RF	1.80**	0.000	20.02**	0.000	56.54**	0.000	4.54	21.86	13.88	20.92	11.9–33.4	0.46	12.90
	IR	1.38*	0.034	13.90**	0.000	63.08**	0.000	4.55	23.39	15.05	21.37	12.5–32.6	0.28	10.80
BY/pt	RF	1.84**	0.012	23.97**	0.000	86.39**	0.000	44.6	92.78	81.43	86.22	58.5–118.5	0.19	5.90
	IR	1.41*	0.025	16.57**	0.000	122.54**	0.000	42.73	106.53	87.45	97.61	63.7–158.6	0.30	5.10
HI	RF	2.41**	0.000	14.40**	0.000	17.69**	0.000	4.17	24.90	18.40	25.18	19.5–31.9	0.66	7.30
	IR	2.10**	0.000	24.70**	0.000	64.13**	0.000	3.70	22.23	16.30	22.26	16.2–27.1	0.55	4.30
SY/plot	RF	2.30**	0.000	22.47**	0.000	36.78**	0.000	8438	982.18	512.10	886.44	516–1164.1	0.61	10.60
	IR	2.22**	0.000	13.63**	0.000	49.15**	0.000	7444.2	1017.98	469.80	963.48	568–1382.8	0.57	6.80
TSW	RF	8.34**	0.000	11.36**	0.000	64.02**	0.000	0.051	4.98	2.96	4.91	3.4–6.6	0.90	7.50
	IR	5.51**	0.000	2.35 ns	0.125	59.90**	0.000	0.06	4.07	2.78	4.30	2.9–5.7	0.85	6.40
Oil content	RF	5.93**	0.000	28.49**	0.000	21.33**	0.000	1.88	38.84	32.69	37.76	32.2–42.1	0.91	8.20
	IR	4.43**	0.000	34.99**	0.000	3.25*	0.045	2.45	38.24	32.78	38.13	32.9–43.9	0.91	7.40
D50%F	RF	9.22**	0.000	14.65**	0.000	22.75**	0.000	4.63	50.76	76.67	49.43	40.5–62.8	0.92	8.90
	IR	6.05**	0.000	12.53**	0.000	80.69**	0.000	1.92	53.60	80.86	52.79	44.2–65.5	0.85	9.60
Days to maturity	RF	9.21**	0.000	42.29**	0.000	60.35**	0.000	3.62	140.27	152.50	137.97	131.8–144.9	0.76	3.90
	IR	2.47**	0.000	37.18**	0.000	74.62**	0.000	4.64	142.10	157.70	141.50	134.2–148.7	0.63	4.50

χ^2^ = Chi square test of significance for environment and genotype × environment effects using -2 loglikelihood ratio test; *PH* Plant height (cm), *PB/pt* Primary branches per plant, *SB/pt* Secondary branches per plant, *MSL* Main shoot length (cm), *SoMS* Total siliquae on main shoot, *SY/pt* Seed yield per plant (g), *BY/pt* Biological yield per plant (g), *HI* Harvest index (%), *SY/plot* Seed yield per plot (g), TSW = 1,000 seed weight (g), D50% F = Days to 50% flowering, **p ≤ 0.01, *p ≤ 0.05 and *ns* non-significant

### Water use efficiency (WUE) and drought tolerance indices

**Fig. 4:**
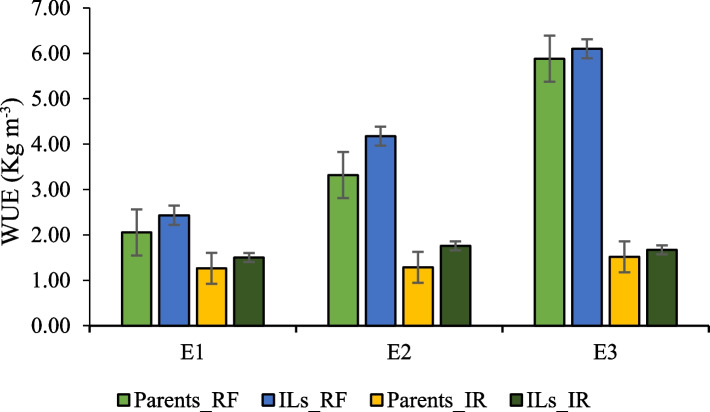
WUE in *B. carinata* derived *B. juncea* introgression lines along with their parents under rainfed (RF) and irrigated (IR) conditions [Environment, E1 = 2018–19 (Delhi); E2 = 2020–21 (Delhi); E3 = 2020–21 (Bharatpur)]

Water use efficiency (WUE) and drought tolerance indices, viz., drought susceptibility index (DSI), drought tolerance index (DTI), tolerance index (TOL) and mean relative performance (MRP) for seed yield (kg/ha) were estimated under three different environments. WUE among the parental lines was observed to vary from 1.23 to 2.88 kg m^−3^, 1.41 to 5.23 kg m^−3^ and 5.06 to 5.70 kg m^−3^ in RE1, RE2 and RE3 environments, respectively, under a moisture deficit stress condition. On the other hand, WUE among ILs ranged from 0.72 to 4.09 kg m^−3^, 1.83 to 6.19 kg m^−3^ and 1.77 to 10.56 kg m^−3^ in RE1, RE2 and RE3 environments, respectively. The highest and lowest values for this trait were recorded for IL94 and IL154 in RE1, IL160 and IL135 in RE2 and IL160 and IL125 in RE3 environments, respectively (Supplementary Table). In all the environments, ILs had a higher WUE than their *B. juncea* parent (Fig. 4). Results also revealed that in all environments, the mean WUE of parents and ILs were higher under rainfed conditions than under irrigated conditions (Fig. 4).

**Fig. 5:**
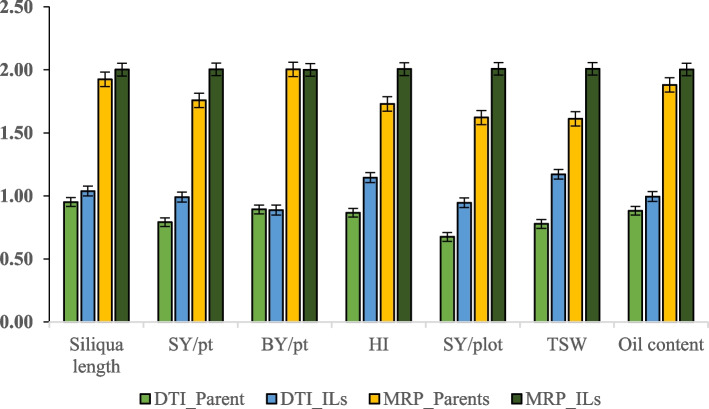
The drought tolerant index (DTI) and mean relative performance (MRP) observed on important seed yield contributing traits in ILs along with parents evaluated under rainfed and irrigated conditions. (*SY/pt* Seed yield/plant, *BY/pt* Biological yield/plant, *HI* Harvest index, *SY/plot* Seed yield/plot, *TSW* 1,000 seed weight)

Important seed yield contributing traits such as siliqua length (cm), seed yield per plant (g), biological yield per plant (g), harvest index, seed yield per plot (g), 1,000 seed weight (g) and oil content (%) exhibited higher DTI and MRP for ILs than their parents (Fig. 5). DTI for seed yield varied from 0.17 to 2.73, 0.18 to 2.21 and 0.18 to 2.12 in ILs, whereas it varied from 0.12 to 1.46, 0.10 to 1.40 and 0.35 to 1.25 in their parents in E1, E2 and E3 environments, respectively (Supplementary Table). Similarly, MRP varied from 0.82 to 2.68, 0.63 to 2.37 and 1.31 to 2.36 in parents, whereas it ranges from 0.92 to 3.68, 0.85 to 2.98 and 0.96 to 3.09 in ILs in E1, E2 and E3 environments, respectively (Supplementary Table). ILs, viz., IL104, IL106, IL119, IL121, IL126, IL128, IL131 and IL135 have a DTI value of > 1 and a MRP value of > 2, indicating their superiority under both rainfed and irrigated conditions across all the environments (Supplementary Table).

### Construction of linkage map

The linkage map was constructed using 5,165 genome-wide SNPs obtained from GBS and grouped into 838 unique bins (Table 2). The linkage map spanned over a length of 1,671.87 cM with an average marker interval of 2.00 cM and an average marker density of 3.09 cM. The number of genetic bins on each chromosome varied from 25 (10A) to 65 (9A) and markers from 159 (10A) to 438 (9A) per chromosome. The shortest chromosome was 4A, which carried 204 markers with a genetic length of 5.21 cM, an average marker interval of 0.12 cM, and a marker density of 39.16 per marker. The 3B chromosome was the longest, with 277 markers, 245.27 cM genetic length and an average marker interval of 6.45 cM. Marker density was found to be lowest in chromosome 3B (1.13 cM per marker) and highest in 4A (39.16 cM per marker), followed by 5B (10.25 cM per marker). The A and B genomes contain 2932 and 2233 markers with a total length of 736.17 cM and 935.70 cM, respectively. Among the two genomes of *B. juncea*, the A genome had a higher marker density (3.98 cM per marker and 56.77% of total markers) than the B genome (2.39 cM per marker and 43.23% of total markers) (Table 2).

**Table 2:** Marker statistics of the linkage map constructed from introgression lines derived from cross between DRMRIJ 31 and BC 4

Chromosome	Number of SNPs	% Markers	Map length (cM)	Bins	Average markers/ bin interval	Marker density
1A	298	5.77	72.50	41	1.77	4.11
2A	194	3.76	56.18	37	1.52	3.45
3A	431	8.34	95.90	49	1.96	4.49
4A	204	3.95	5.21	42	0.12	39.16
5A	296	5.73	73.10	48	1.52	4.05
6A	405	7.84	123.00	52	2.37	3.29
7A	267	5.17	104.65	47	2.23	2.55
8A	240	4.65	49.58	58	0.85	4.84
9A	438	8.48	90.13	65	1.39	4.86
10A	159	3.08	65.92	25	2.64	2.41
1B	220	4.26	120.61	39	3.09	1.82
2B	229	4.43	91.72	45	2.04	2.50
3B	277	5.36	245.27	38	6.45	1.13
4B	311	6.02	83.92	65	1.29	3.71
5B	318	6.16	31.02	50	0.62	10.25
6B	250	4.84	181.72	43	4.23	1.38
7B	331	6.41	95.22	60	1.59	3.48
8B	297	5.75	86.22	34	2.54	3.44
A genome	2932	56.77	736.17	464	1.59	3.98
B genome	2233	43.23	935.70	374	2.50	2.39
Total	5165		1671.87	838	2.00	3.09

### Mapping of QTLs for agro-morphological traits under drought conditions

In three different environments, namely 2018–19 (Delhi; E1), 2020–21 (Delhi; E2), and 2020–21 crop season (Bharatpur; E3), created under moisture deficiency stress conditions, a total of 24 additive QTLs linked with seed yield and its contributing attributes were discovered (Table 3). These QTLs were distributed over eleven chromosomes (3A, 7A, 8A, 9A, 10A, 1B, 2B, 3B, 5B, 6B and 8B) and explained about 5.49 to 20.65% of the total phenotypic variation (PV). Among 24 QTLs, 14 (58%) loci carried alleles from the *B. carinata* parent (BC 4) for increasing phenotypic values and to withstand moisture deficit stress. While the remaining 10 (42%) loci carried alleles from the *B. juncea* parent (Table 3).

**Table 3:** Significant QTLs detected using high-density linkage map for yield contributing traits in ILs evaluated across the environments created under rainfed situations

Trait	QTL	Env	Chr	Pos	Flanking Markers	LOD	PVE (%)	Add	Marker interval (cM)
Plant height	*qPH.2B.1*	RE1	2B	10	*2B_3291748–2B_1636089*	3.26	16.38	-9.88	2.5–15.5
	*qPH.3B.1*	RE2	3B	119	*3B_19151635–3B_18990496*	2.88	11.55	6.84	116.5–119.5
	*qPH.9A.1*	RE3	9A	72	*9A_45604301–9A_3104692*	2.78	15.62	13.52	71.5–73.5
Secondary branches/plant	*qSB.3B.1*	RE1	3B	58	*3B_65754767–3B_63675862*	3.05	15.66	-4.67	57.5–58.5
	*qSB.7A.1*	RE2	7A	80	*7A_6835604–7A_7834496*	3.10	5.49	3.01	76.5–82.5
	*qSB.10A.1*		10A	49	*10A_17921440–10A_847901*	2.95	5.65	2.92	47.5–49.5
Main shoot length	*qMSL.3B.1*	RE1	3B	103	*3B_9101086–3B_22690875*	3.19	16.17	-5.28	96.5–111.5
Siliqua length	*qSL.2B.1*	RE1	2B	64	*2B_65115276–2B_64577224*	4.32	20.65	0.25	62.5–64.5
	*qSL.2B.1*	RE2	2B	64	*2B_65115276–2B_64577224*	2.51	12.96	0.25	62.5–64.5
	*qSL.8A.1*	RE3	8A	27	*8A_3395566–8A_3395574*	2.63	15.07	0.51	26.5–28.5
Seeds/ silique	*qSS.9A.1*	RE1	9A	24	*9A_38513927–9A_5962669*	2.82	18.21	2.64	23.5–24.5
	*qSS.9A.1*	RE3	9A	24	*9A_38513927–9A_5962669*	2.81	13.89	3.32	23.5–24.5
Seed yield/ plant	*qSY.3B.1*	RE3	3B	100	*3B_9101086–3B_22690875*	2.74	14.15	-1.74	94.5–103.5
	*qSY.3B.2*	RE2	3B	111	*3B_22690875–3B_19151635*	3.39	18.34	-2.55	104.5–115.5
Biological yield/plant	*qBY.3A.1*	RE2	3A	87	*3A_2676786–3A_2045625*	4.36	8.59	16.55	85.5–87.5
	*qBY.3B.1*	RE2	3B	20	*3B_60523215–3B_58467664*	3.08	5.53	8.26	16.5–21.5
	*qBY.3B.2*	RE3	3B	101	*3B_9101086–3B_22690875*	3.03	11.91	-5.44	96.5–104.5
	*qBY.3B.2*	RE2	3B	104	*3B_9101086–3B_22690875*	5.52	10.75	-8.96	99.5–110.5
1,000 seed weight	*qTSW.3B.1*	RE3	3B	237	*3B_59798024–3B_16518040*	2.70	13.66	-0.34	229.5–237.5
	*qTSW.3B.2*	RE1	3B	243	*3B_16490510–3B_15005559*	3.73	14.58	-0.47	238.5–245
	*qTSW.3B.2*	RE2	3B	242	*3B_16490510–3B_15005559*	2.98	14.40	-0.31	237.5–245
	*qTSW.6B.1*	RE2	6B	120	*6B_17473921–6B_17473911*	2.83	11.78	0.24	111.5–126.5
Oil content	*qOIL.8A.1*	RE1	8A	47	*8A_16951000–8A_1068464*	2.58	11.49	-1.11	44.5–49
Days to 50% flowering	*qDF.5B.1*	RE1	5B	9	*5B_51917617–5B_48146581*	3.48	7.42	10.31	7.5–10.5
	*qDF.1B.1*	RE2	1B	71	*1B_56351113–1B_30563611*	3.82	17.84	2.57	68.5–72.5
	*qDF.3B.1*	RE3	3B	116	*3B_22690875–3B_19151635*	3.13	15.07	1.60	110.5–119.5
Days to maturity	*qDM.8B.1*	RE2	8B	22	*8B_69572249–8B_69878598*	2.89	16.35	1.95	20.5–22.5
	*qDM.2B.1*	RE3	2B	53	*2B_58202892–2B_64284809*	3.36	12.41	-4.85	48.5–56.5

Where, *Env* Environment, *Chr* Chromosome, *Pos* Chromosome position (cM) of the QTL, *LOD* Logarithm of the odds, *PVE* Phenotypic variance explained (%); *Add* Additive effect (positive values of the additive effect indicated that alleles from parent ‘BC 4’ were in the direction of increasing trait score and negative indicated that alleles from parent ‘DRMRIJ 31’ were in the direction of increasing trait score); RE1 = 2018–19; RE2 = 2020–21-Delhi; RE3 = 2020–21-Bharatpur

Three additive effect QTLs were identified, one in each environment for plant height, explaining PV from 11.55% to 16.38% and distributed over three chromosomes (9A, 2B and 3B). For secondary branches per plant, three QTLs were detected on three different chromosomes (7A, 10A and 3B) and explained 5.49 to 15.66% PV. A QTL regulating main shoot length under moisture deficit stress was identified to exhibit high PV (16.17%). For siliqua length two major QTLs, viz., *qSL.2B.1* on chromosome 2B and *qSL.8A.1* on chromosome 8A were detected to explain PV of 12.96 and 20.65%, respectively. The QTL *qSL.2B.1*, flanked by markers *2B_65115276* and *2B_64577224*, was found to regulate siliqua length in two of the three environments. All the QTLs associated with siliqua length observed positive additive effects, indicating the contribution of *B. carinata* parent BC 4 to increase siliqua length under moisture deficit stress conditions (Table 3).

For seeds per siliqua, a major QTL (*qSS.9A.1)*, which is flanked by markers *9A_38513927* and *9A_5962669*, was detected in two different environments and explained PV up to 18.21%. Two QTLs on chromosome 3B were found to be associated with seed yield per plant under moisture deficit stress conditions, and these explained PV ranging from 14.15 to 18.34% (Table 3). Three QTLs were discovered to be associated with biological yield per plant; they were present on chromosomes 3A and 3B and explained PV ranging from 5.53 to 11.91% under moisture deficit stress conditions. A QTL flanked by markers *3B_9101086* and *3B_22690875* (*qBY.3B.2*) was detected to explain high PV in two different environments, whereas, another QTL on chromosome 8A explained 11.49% variation for oil content. Three QTLs, viz., *qTSW.3B.1*, *qTSW.3B.2* and *qTSW.6B.1*, were found to regulate 1,000-seed weight (g). Two of these were found to be located on chromosome 3B and the other on chromosome 6B, explaining PV from 11.78 to 14.58%, respectively. Another QTL for 1000-seed weight (*qTSW.3B.2*) flanked by *3B_16490510* and *3B_15005559* markers was found to explain 14.40 to 14.58% PV in two environments. For days to 50% flowering, three QTLs were detected, one in each environment, explaining PV from 7.42 to 17.84% and were located on three different chromosomes (1B, 3B and 5B). QTLs viz., *qDM.8B.1* and *qDM.8B.1* were reported to be associated with shorter maturity duration under moisture deficit stress in E2 and E3 environments, explaining 12.41 and 16.35% of PVs, respectively (Table 3).

Several QTLs with a wide range of QTL × environment interactions were detected across the environments under moisture deficit stress conditions (Table 4). For plant height, QTL detected on chromosome 2B (*qPH.2B.1*) had a high Logarithm of Odds (LOD) score for additive effect [LOD (A)] and a small LOD score for additive × environment effect [LOD (A × E)], which indicated weak QTL-environment interactions. QTL, *qPH.3B.1* exhibited a high LOD (A × E) and low LOD (A) values, implying significant interactions between the QTL and the environment. QTLs for primary and secondary branches per plant showed very high QTL × environment interactions, which is evident by the small value of LOD (A) and high LOD (A × E). In the case of main shoot length, out of three QTLs detected, one on 3B (*qMSL.3B.2)* exhibited very strong QTL × environment interactions. All the QTLs identified for siliqua length, seeds per siliqua, 1000-seed weight and oil content displayed high values for LOD (A) and small LOD (A × E) values. The QTLs for seed yield per plant and biological yield per plant exhibited medium-to-high QTL × environment interactions. For days to 50% flowering, all identified QTLs showed small QTL × environment interactions. Only one QTL present on chromosome 8B (*qDM.8B.1*), explaining variation for days to maturity under moisture deficit stress conditions, had strong QTL × environment interactions, while the rest were responsible for controlling variation for this trait only in one or the other environments (Table 4).

**Table 4:** QTL x environment interaction estimated for yield and its contributing traits evaluated across the environments created under rainfed situation

Trait	QTL	Chr	Pos	Marker interval	LOD	LOD (A)	LOD (A × E)	PVE	PVE (A)	PVE (A × E)	Add	Confidence interval (cM)
Plant height	*qPH.2B.1*	2B	11	*2B_3291748–2B_1636089*	3.56	2.62	0.94	11.42	6.88	4.54	-4.31	3.5–15.5
	*qPH.3B.1*	3B	119	*3B_19151635–3B_18990496*	3.58	0.70	2.87	10.66	2.83	7.82	2.05	116.5–119.5
Primary branches/plant	*qPB.3B.1*	3B	69	*3B_8677419–3B_8677464*	3.05	0.04	3.01	28.67	0.53	28.14	0.03	67.5–73.5
Secondary Branches/plant	*qSB.3B.1*	3B	27	*3B_15825638–3B_12057245*	3.68	1.87	1.81	19.55	9.30	10.25	0.57	26.5–29.5
	*qSB.3B.2*	3B	58	*3B_65754767–3B_63675862*	4.3	0.04	4.26	17.41	0.05	17.35	-0.13	57.5–58.5
	*qSB.5A.1*	5A	48	*5A_7330303–5A_8808015*	3.45	0.69	2.76	17.36	3.82	13.54	0.96	47.5–49.5
Main shoot length	*qMSL.3B.1*	3B	104	*3B_9101086–3B_22690875*	4.05	3.40	0.66	10.53	7.80	2.70	-2.79	98.5–110.5
	*qMSL.3B.2*	3B	224	*3B_59798024–3B_16518040*	3.15	0.01	3.14	11.00	1.10	9.90	-0.08	218.5–237.5
	*qMSL.6A.1*	6A	122	*6A_38048376–6A_39119449*	4.76	4.11	0.65	10.20	9.21	0.99	3.04	116.5–122
Siliqua length	*qSL.2B.1*	2B	64	*2B_65115276–2B_64577224*	8.82	8.16	0.66	10.04	10.00	0.04	0.24	63.5–64.5
	*qSL.7A.1*	7A	26	*7A_22615478–7A_17513851*	4.57	4.38	0.19	6.23	6.19	0.04	0.57	24.5–27.5
	*qSL.8A.1*	8A	22	*8A_22681841–8A_2967198*	4.00	3.34	0.67	3.71	3.50	0.21	0.29	20.5–23.5
	*qSL.8A.2*	8A	27	*8A_3395566–8A_3395574*	4.06	3.82	0.24	4.26	3.55	0.70	0.32	26.5–27.5
	*qSL.9A.1*	9A	24	*9A_38513927–9A_5962669*	5.33	4.83	0.50	6.11	6.08	0.03	0.61	23.5–24.5
	*qSL.1B.1*	1B	106	*1B_57398572–1B_2361130*	3.54	3.26	0.28	2.65	2.65	0.00	0.19	104.5–109.5
	*qSL.3B.1*	3B	112	*3B_22690875–3B_19151635*	3.20	3.00	0.20	2.59	2.46	0.13	-0.13	100.5–121.5
	*qSL.3B.2*	3B	238	*3B_16490510–3B_15005559*	4.55	4.51	0.04	3.75	3.59	0.16	-0.20	232.5–240.5
	*qSL.4B.1*	4B	57	*4B_57678828–4B_6261023*	4.02	3.37	0.66	2.90	2.86	0.05	0.14	50.5–60.5
	*qSL.6B.1*	6B	150	*6B_3716052–6B_7548078*	3.38	3.03	0.36	2.80	2.43	0.37	0.13	145.5–151.5
Seeds/siliqua	*qSS.9A.1*	9A	24	*9A_38513927–9A_5962669*	6.95	6.8	0.15	10.82	10.42	0.40	2.53	23.5–24.5
	*qSS.7A.1*	7A	24	*7A_22615478–7A_17513851*	3.65	3.46	0.19	4.31	4.26	0.04	1.11	18.5–27.5
	*qSS.8B.1*	8B	86	*8B_9276009–8B_3535762*	3.12	3.10	0.02	3.25	3.20	0.05	-0.45	74.5–86.0
Seed yield/plant	*qSY.3B.2*	3B	108	*3B_22690875–3B_19151635*	6.25	3.84	2.41	7.88	7.57	0.31	-1.79	104.5–114.5
Biological yield/ plant	*qBY.3A.1*	3A	87	*3A_2676786–3A_2045625*	4.52	0.59	3.93	4.37	1.30	3.08	4.80	85.5–87.5
	*qBY.3B.1*	3B	20	*3B_60523215–3B_58467664*	3.21	1.01	2.21	3.59	2.20	1.38	3.96	16.5–21.5
	*qBY.3B.2*	3B	104	*3B_9101086–3B_22690875*	8.41	3.35	5.07	8.74	7.49	1.25	-5.68	99.5–109.5
1,000 seed weight	*qTSW.3B.2*	3B	243	*3B_16490510–3B_15005559*	6.75	5.75	1.00	13.73	10.41	3.32	-0.26	239.5–245.5
	*qTSW.6B.1*	6B	120	*6B_17473921–6B_17473911*	5.47	5.08	0.39	10.24	9.40	0.84	0.20	113.5–125.5
	*qTSW.3A.1*	3A	61	*3A_22246594–3A_10271312*	3.38	3.32	0.06	6.01	5.74	0.27	0.19	58.5–65.5
	*qTSW.5A.1*	5A	1	*5A_29328695–5A_29019992*	3.31	2.49	0.82	5.44	5.39	0.05	-0.75	0–1.5
Oil content (%)	*qOL.3A.1*	3A	47	*3A_2645497–3A_22246594*	4.54	4.35	0.19	4.73	4.67	0.07	0.78	40.5–52.5
	*qOL.8A.1*	8A	48	*8A_16951000–8A_1068464*	5.67	5.46	0.21	6.12	5.87	0.25	-0.85	45.5–49
	*qOL.10A.1*	10A	28	*10A_18208177–10A_17921440*	3.09	2.74	0.35	3.08	2.89	0.19	0.58	14.5–42.5
	*qOL.2B.1*	2B	40	*2B_54313137–2B_54313158*	3.09	3.04	0.05	3.53	3.42	0.11	-1.91	39.5–41.5
	*qOL.5B.1*	5B	28	*5B_291261–5B_54290*	4.33	4.17	0.16	4.46	4.39	0.06	-1.49	27.5–28.5
	*qOL.6B.1*	6B	181	*6B_8989606–6B_9051954*	3.82	3.58	0.24	3.76	3.68	0.07	-0.85	179.5–181
	*qOL.7B.1*	7B	23	*7B_34135672–7B_48748636*	3.08	2.57	0.51	2.71	2.62	0.10	-0.75	21.5–25.5
	*qOL.7B.2*	7B	86	*7B_36243896–7B_37982990*	3.01	2.36	0.66	2.79	2.67	0.12	-1.03	85.5–86.5
Days to 50% flowering	*qDF.3A.1*	3A	32	*3A_2645497–3A_22246594*	3.62	3.38	0.23	6.30	4.94	1.36	1.19	28.5–40.5
	*qDF.1B.1*	1B	71	*1B_56351113–1B_30563611*	5.26	3.05	2.21	6.10	5.63	0.47	2.15	69.5–72.5
	*qDF.2B.1*	2B	46	*2B_58470758–2B_58202895*	4.77	4.14	0.64	7.82	6.52	1.29	-2.07	45.5–46.5
	*qDF.3B.1*	3B	118	*3B_19151635–3B_18990496*	5.13	4.13	0.99	7.60	6.28	1.31	1.41	114.5–119.5
	*qDF.3B.2*	3B	234	*3B_59798024–3B_16518040*	4.02	3.66	0.36	6.59	5.40	1.19	1.49	223.5–240.5
	*qDF.5B.1*	5B	8	*5B_51917617–5B_48146581*	3.23	2.78	0.45	6.31	3.76	2.55	2.64	7.5–10.5
	*qDF.6B.1*	6B	77	*6B_18597429–6B_13740232*	3.63	2.73	0.90	4.97	4.14	0.84	-1.17	64.5–80.5
	*qDF.7B.1*	7B	49	*7B_49935609–7B_49542209*	4.00	3.66	0.35	6.93	5.49	1.44	-1.23	47.5–56.5
Days to maturity	*qDM.3B.1*	3B	119	*3B_19151635–3B_18990496*	4.34	4.22	0.12	12.21	8.74	3.47	1.21	116.5–119.5
	*qDM.2B.1*	2B	48	*2B_58202892–2B_64284809*	3.30	3.20	0.10	8.38	5.65	2.74	-1.28	45.5–56.5
	*qDM.7B.1*	7B	49	*7B_49935609–7B_49542209*	3.87	3.44	0.43	7.49	6.64	0.86	-0.98	47.5–55.5
	*qDM.8B.1*	8B	21	*8B_69572176–8B_69572249*	3.26	1.05	2.22	4.32	2.08	2.23	0.82	20.5–22.5

Where, *Chr* Chromosome, *Pos* Chromosome position (cM) of the QTL, *LOD* Logarithm of the odds, *LOD(A)* LOD score for additive effects, *LOD (A* × *E)* LOD score for additive by environment effects, PVE, PVE (A) and PVE (A × E) is Phenotypic variance explained by QTL, additive effects and additive × environment effects, respectively

### Mapping of QTLs for water use efficiency and drought tolerance indices

A total of five additive QTLs associated with different WUE and drought tolerance indices were identified in three environments under moisture deficit stress conditions (Table 5). These were located on four different chromosomes (7A, 2B, 7B and 8B) and explained PV ranging from 5.42 to 24.18%. Out of these five QTLs, three had a positive additive effect, which indicated that these loci carry alleles from the *B. carinata* parent (BC 4) and are responsible for improving drought tolerance in the ILs. The rest of the two QTLs identified in these ILs were inherited from *B. juncea* cultivar DRMRIJ 31.

**Table 5:** Significant QTLs detected for different drought tolerance indices under rainfed and irrigated conditions across three environments

Trait	QTL	Env	Chr	Pos	Marker interval	LOD	PVE (%)	Additive effect	Confidence interval (cM)
WUE	*qWUE.7B.1*	E3	7B	14	*7B_30548045–7B_870706*	3.89	13.40	0.32	13.5–14.5
DTI	*qDTI.2B.1*	E1	2B	9	*2B_3291748–2B_1636089*	3.53	12.51	0.22	0–14.5
	*qDTI.7A.1*	E3	7A	28	*7A_27314562–7A_27853328*	3.51	24.18	0.38	27.5–28.5
MRP	*qMRP.2B.1*	E1	2B	8	*2B_3291748–2B_1636089*	3.61	12.63	-0.24	0–13.5
	*qMRP.8B.1*	E2	8B	14	*8B_35192556–8B_68780330*	3.96	5.42	-0.22	13.5–17.5

*Env* Environment, *Chr* Chromosome, *Pos* Chromosome position (cM) of the QTL, *LOD* logarithm of the odds, *WUE* Water use efficiency (kg m^−3^) under rainfed condition, *DTI* Drought tolerance index, and *MRP* Mean relative performance

A QTL (*qWUE.7B.1*) with flanking markers *7B_30548045* and *7B_870706* was found to improve WUE under moisture deficit stress conditions and explained up-to 13.40% PV. Two QTLs, viz., *qDTI.2B.1* and *qDTI.7A.1*, were reported to control the DTI and explained high PV (12.51 and 24.18%, respectively). Similarly, two QTLs (*qMRP.2B.1* and *qMRP.8B.1*) were reported to explain 12.63 and 5.42% PV for MRP, respectively (Table 5). Another QTL (*qDTI.2B.1*) located on the 2B chromosome, flanked by markers *2B_3291748* and *2B_1636089*, was identified to explain more than 10% PV for DTI and MRP traits (Table 5).

Significant QTL × environment interactions were also observed for QTLs explaining WUE and drought tolerance indices across the environments (Table 6). A total of 16 QTLs were reported to influence WUE under moisture deficit stress and drought tolerance indices. Six QTLs responsible for improving WUE in this study were identified on three different chromosomes, and three of them (*qWUE.7A.1*, *qWUE.7A.2* and *qWUE.3B.1*) had higher LOD (A) than LOD (A × E), indicating small QTL × environment interactions. The other three QTLs (*qWUE.7B.1*, *qWUE.7B.2* and *qWUE.7B.3*), on the other hand, have significant QTL × environment interactions. All five QTLs responsible for improved DTI exhibited higher LOD (A) than LOD (A × E), suggesting smaller QTL × environment interactions. Four QTLs associated with higher MRP, namely *qMRP.7A.1*, *qMRP.1B.1*, *qMRP.2B.1* and *qMRP.3B.1*, had higher LOD (A) than LOD (A × E), and had small QTL × environment interactions. Higher QTL × environment interaction was also observed for *qMRP.8B.1* QTL (Table 6).

**Table 6:** QTL × environment interactions affecting different drought tolerance indices under rainfed and irrigated conditions across three environments

Trait	QTL	Chr	Pos	Marker interval	LOD	LOD (A)	LOD (A × E)	PVE	PVE (A)	PVE (A × E)	Additive effect	Confidence interval (cM)
WUE	*qWUE.7A.1*	7A	13	*7A_14916577–7A_22542484*	3.26	2.83	0.43	5.61	5.37	0.24	0.12	11.5–18.5
	*qWUE.7A.2*	7A	28	*7A_27314562–7A_27853328*	4.39	3.76	0.63	9.21	8.30	0.90	0.30	27.5–28.5
	*qWUE.3B.1*	3B	119	*3B_19151635–3B_18990496*	3.42	2.22	1.20	6.24	4.20	2.04	-0.08	114.5–123.5
	*qWUE.7B.1*	7B	0	*7B_38657519–7B_40804516*	3.11	0.98	2.13	5.66	1.50	4.16	-0.05	0–5.5.0
	*qWUE.7B.2*	7B	14	*7B_30548045–7B_870706*	3.31	1.21	2.10	5.83	1.78	4.04	0.07	13.5–14.5
	*qWUE.7B.3*	7B	62	*7B_49573087–7B_46012400*	3.29	1.18	2.11	6.01	1.93	4.08	-0.06	59.5–67.5
DTI	*qDTI.7A.1*	7A	28	*7A_27314562–7A_27853328*	4.77	3.64	1.13	7.44	6.75	0.69	0.27	27.5–28.5
	*qDTI.1B.1*	1B	98	*1B_47952601–1B_2982196*	3.12	2.8	0.32	5.78	5.57	0.21	0.19	97.5–98.5
	*qDTI.2B.1*	2B	10	*2B_3291748–2B_1636089*	3.32	2.26	1.06	7.77	4.36	3.41	0.09	0–14.5
	*qDTI.4B.1*	4B	5	*4B_6040423–4B_52400595*	3.22	2.04	1.18	6.02	3.90	2.12	-0.12	3.5–5.5
	*qDTI.6B.1*	6B	157	*6B_5822839–6B_4974945*	3.28	1.94	1.34	5.23	3.74	1.48	-0.09	155.5–157.5
MRP	*qMRP.7A.1*	7A	42	*7A_27686964–7A_26280398*	3.61	2.95	0.66	6.19	5.46	0.73	0.11	28.5–49.5
	*qMRP.1B.1*	1B	98	*1B_47952601–1B_2982196*	3.15	2.68	0.47	5.43	5.03	0.40	0.18	97.5–98.5
	*qMRP.2B.1*	2B	10	*2B_3291748–2B_1636089*	3.29	2.2	1.09	7.53	3.93	3.60	-0.09	0–14.5
	*qMRP.3B.1*	3B	120	*3B_19012909–3B_18718709*	3.59	2.81	0.78	5.42	5.16	0.26	-0.10	113.5–125.5
	*qMRP.8B.1*	8B	14	*8B_35192556–8B_68780330*	3.76	1.63	2.13	5.86	2.88	2.98	-0.09	13.5–17.5

*Chr*  =  Chromosome, *Pos*  =  Chromosome position (cM) of the QTL, *LOD*  =  Logarithm of the odds, *LOD (A)* LOD score for additive effects, *LOD (A × E)*  LOD score for additive by environment effects, PVE, PVE (A) and PVE (A × E) is Phenotypic variance explained by QTL, additive effects and additive × environment effects, respectively *WUE*  Water use efficiency (kg m^−3^) under rainfed condition; *DTI* Drought tolerance index; and *MRP* Mean relative perform

### Identification of co-localized QTLs and candidate genes in the QTL regions

**Fig. 6:**
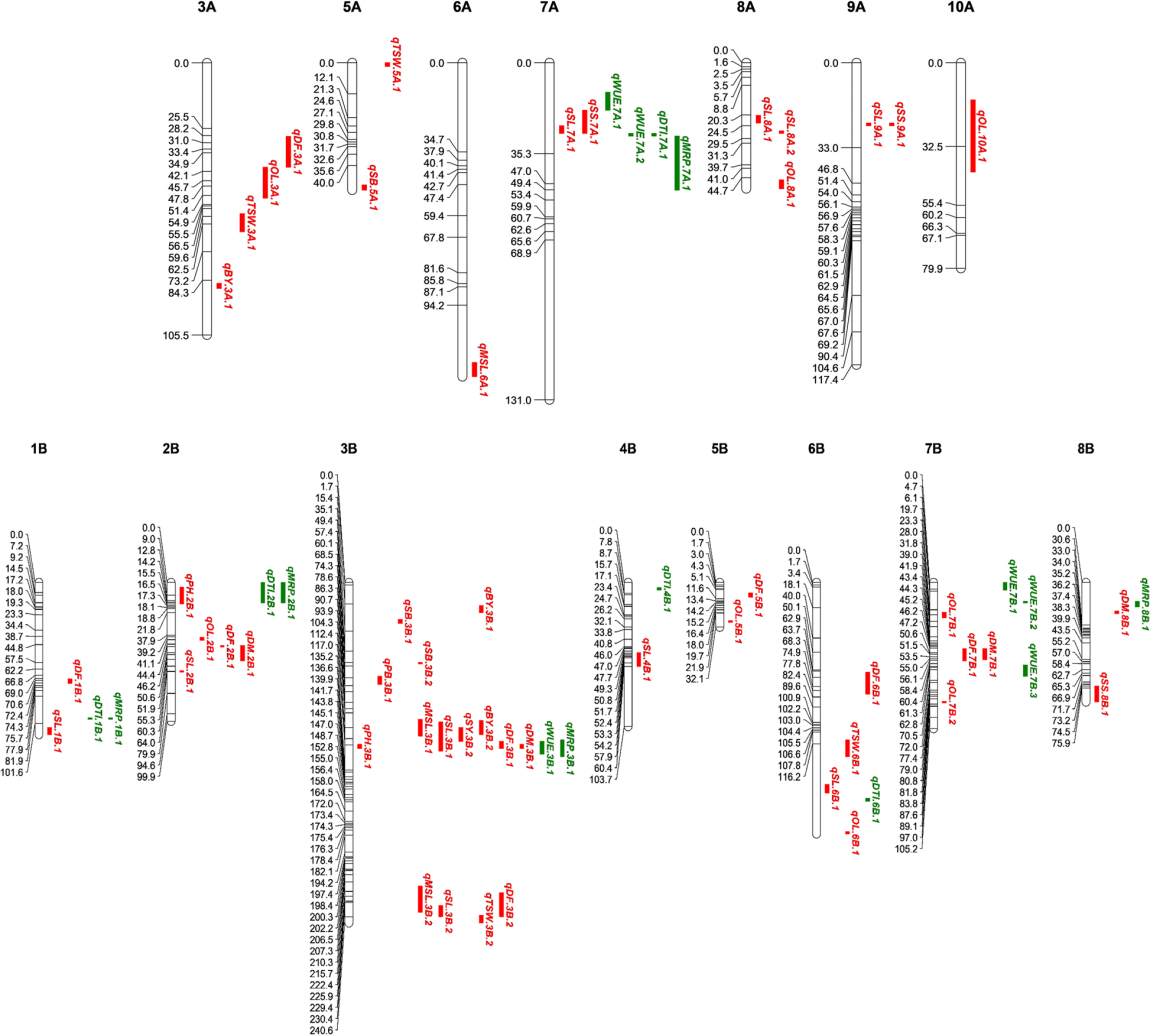
The linkage map depicting QTLs for agro-morphological traits, water use efficiency and drought tolerance indices across the environments

Analysis was performed for the identification of two or more co-localized QTLs, at the same position in the genome, governing agro-morphological traits, WUE and drought tolerance indices under moisture deficit stress conditions (Table 7; Fig. 6). Four QTLs, viz*.*, *qSS.7A.1*, *qSL.7A.1*, *qWUE.7A.2* and *qDTI.7A.1* were found to be located together in the small confidence interval on chromosome 7A. Two QTLs (*qSL.9A.1* and *qSS.9A.1*) controlling siliqua length and seeds per siliqua**,** respectively, were found co-localized on the 9A chromosome. Two QTLs (*qDTI.1B.1* and *qMRP.1B.*1) on 1B and two (*qDF.2B.1* and *qDM.2B.1*) on the 2B chromosome were detected at the same position in the genome (Fig. 6). The hotspot carrying eight QTLs, viz., *qBY.3B.2*, *qMSL.3B.1*, *qSY.3B.1*, *qSL.3B.1*, *qDF.3B.1*, *qPH.3B.1*, *qDM.3B.1* and *qWUE.3B.1* was identified on chromosome 3B in a small confidence interval. Furthermore, QTLs *qDF.7B.1* and *qDM.7B.1* were also reported to be located at the same position on chromosome 7B (Table 7; Fig. 6). To make any *B. juncea* line tolerant to moisture deficit stress, QTL hotspots identified in the present study can be further transferred from ILs through MAS.

**Table 7:** Co-localized QTLs for one or more traits and position of the candidate genes in the identified QTL regions

Trait	QTL	Marker interval	Position (cM)	Confidence interval (cM)	LOD	PVE (%)	Candidate genes reported
Main shoot length	*qMSL.6A.1*	*6A_38048376–6A_39119449*	122	116.5–122	4.76	10.2	*SOS2 like* gene [50]; *NPR1* [51]
Seeds per siliqua	*qSS.7A.1*	*7A_22615478–7A_17513851*	24	18.5–27.5	3.65	4.31	*FAE1 KCS* [52]
Siliqua length	*qSL.7A.1*		26	24.5–27.5	4.57	6.23	
Water use efficiency	*qWUE.7A.2*	*7A_27314562–7A_27853328*	28	27.5–28.5	4.39	10.21	*HOT5* [53]; *DNAJA1* [54]; *NIA1* [55]
Drought tolerance index	*qDTI.7A.1*		28	27.5–28.5	4.77	7.44	
Mean relative performance	*qMRP.7A.1*	*7A_27686964–7A_26280398*	42	28.5–49.5	3.61	6.19	*BRI1* [56]
Siliqua length	*qSL.9A.1*	*9A_38513927–9A_5962669*	24	23.5–24.5	5.33	6.11	*RF21*, y*cf2* [57]
Seeds/siliqua	*qSS.9A.1*		24	23.5–24.5	6.95	10.82	
Drought tolerance index	*qDTI.1B.1*	*1B_47952601–1B_2982196*	98	97.5–98.5	3.12	5.78	–
Mean relative performance	*qMRP.1B.1*		98	97.5–98.5	3.15	5.43	
Days to 50% flowering	*qDF.2B.1*	*2B_58470758–2B_58202895*	46	45.5–46.5	4.77	7.82	–
Days to maturity	*qDM.2B.1*		48	45.5–56.5	3.30	8.38	
Secondary branches/plant	*qSB.3B.1*	*3B_65754767–3B_63675862*	58	57.5–58.5	4.30	17.40	*WRKY–33* [58]
Biological yield/plant	*qBY.3B.2*	*3B_9101086–3B_22690875*	104	99.5–109.5	8.41	8.74	*PAL* [59]; *SAMS2* [60–62]
Main shoot length	*qMSL.3B.1*		104	98.5–110.5	4.05	10.53	
Seed yield/plant	*qSY.3B.2*	*3B_22690875–3B_19151635*	108	104.5–114.5	6.25	7.88	*SOS2* like gene [50]
Siliqua length	*qSL.3B.1*		112	100.5–121.5	3.20	2.59	
Days to 50% flowering	*qDF.3B.1*	*3B_19151635–3B_18990496*	119	114.5–119.5	5.13	7.60	*orf147* (mitochondria)
Plant height	*qPH.3B.1*		119	116.5–119.5	3.58	10.66	
Days to maturity	*qDM.3B.1*		119	116.5–119.5	4.34	12.21	
Water use efficiency	*qWUE.3B.1*		119	114.5–123.5	3.42	6.24	
Siliqua length	*qSL.3B.2*	*3B_16490510–3B_15005559*	238	232.5–240.5	4.55	3.75	*MAPK3* [63]
1,000 seed weight	*qTSW.3B.2*		243	239.5–245	6.75	13.73	
Drought tolerance index	*qDTI.6B.1*	*6B_5822839–6B_4974945*	157	155.5–157.5	3.28	5.23	*SOS2* [50]; *ERF* [64]; *BRI1* [56]; *WRR1* [65]
Water use efficiency	*qWUE.7B.1*	*7B_38657519–7B_40804516*	0	0–5.5	3.11	5.66	Sucrose synthase 09 (*SUS*) [66]
Days to 50% flowering	*qDF.7B.1*	*7B_49935609–7B_49542209*	49	47.5–56.5	4.00	6.93	–
Days to maturity	*qDM.7B.1*		49	47.5–55.5	3.87	7.49

The search for candidate genes in the different QTL regions through in silico analysis on *B. juncea* chromosomes identified a total of seventeen genes in Indian mustard with their orthologs in *Arabidopsis thaliana* (Table 7). *SOS2like* and *NPR1* genes were identified in the *qMSL*.*6A.1* QTL region, while *qSS.7A.1* and *qSL.7A.1* carried the *FAE1 KCS* gene. Three candidate genes, viz., *HOT5*, *DNAJA1* and *NIA1*, were reported in the *qWUE.7A.2* and *qDTI.7A.1* QTL regions. However, QTL *qMRP.7A.1* identified to govern MRP under moisture deficit stress conditions, harbored the *BRI1* (brassinosteroid insensitive 1) gene in the same chromosomal region. The genes *RF21* and y*cf2* were identified to be co-localized in the genomic region of QTLs *qSL.9A.1* and *qSS.9A.1*, whereas genes *PAL and SAMS2* were detected in the *qSB.3B.1*, *qBY.3B.2* and *qMSL.3B.1* QTL regions*.* Furthermore, QTL *qSB.3B.1* harbors the *WRKY-33* gene on the 3B chromosome (Table 7).

Similarly, the QTL responsible for higher DTI (*qDTI.6B.1)* carried candidate genes *SOS2like*, *ERFI*, *BRI* and *WRR1* in the same genomic region, whereas the *MAPK3* gene was co-localized with QTLs *qSL.3B.2* and *qTSW.3B.1*. The gene *SUS* (sucrose synthase 09) was found to be located within *qWUE.7B.*1, a QTL identified to improve WUE under moisture deficit stress conditions (Table 7). The association among identified QTLs and already reported candidate genes that are directly or indirectly involved in the pathways associated with drought tolerance in plants was established in this study. Further, the QTLs carrying putative genes in the same genomic region may even be considered as candidate loci in the future studies.

## Discussion

Interspecific hybridization is a prominent method for generating selectable genetic variation in cultivated *Brassica* species [67]. *B. carinata* being tolerant to various abiotic stresses, including drought, is most suitable for broadening the genetic base of other Brassicas [23, 33]. Introgression lines, carrying genomic segments from an alien species into the genetic background of cultivated genotype(s), are highly desirable for the identification and mapping of novel genes/QTLs. Simultaneously, such efforts provide valuable insights into the genetic basis of drought tolerance. The present study was, thus, aimed to develop *B. carinata*-derived *B. juncea* ILs possessing drought tolerance/improved WUE under water stress conditions and elucidating responsive QTLs/genes.

The existence of significant differences (*P* < 0.001) among the ILs for all agro-morphological traits under both rainfed and irrigated conditions indicated the wide range of variability created for these traits, as reported in different environments (Table 1). Furthermore, the pooled analysis revealed significant genotype-by-environment (G × E) interactions for all the traits studied, suggesting the influence of the environment on the expression of these traits. Notably, under both rainfed and irrigated conditions, a wide range of variation along with the recovery of transgressive segregants was observed for nearly all the agro-morphological traits (Table 1), indicating the distribution of desirable alleles in both parents.

### Water use efficiency and drought tolerance indices

In general, higher values of WUE were observed under rainfed conditions than in irrigated conditions and ILs had higher WUE than their parents in both test environments (Fig. 4). Associations among the drought tolerance indices suggested the usefulness of DTI and MRP in the indirect selection of genotypes suitable for drought tolerance in *B. carinata*-derived *B. juncea* lines [32]. Therefore, in the present study, DTI and MRP were calculated from the average values of ILs and parents. Several ILs exhibited high DTI (> 1) and MRP (> 2) values, indicating their superiority in both rainfed and irrigated conditions across all environments (Supplementary Table). Furthermore, seed yield contributing traits such as siliqua length, seed yield per plant, biological yield per plant, harvest index, seed yield per plot, 1000-seed weight and oil content observed higher DTI and MRP for ILs than their respective parents (Fig. 5), implying that improved WUE in these ILs might be due to the complementation of beneficial alleles [32]. These ILs have the potential to become cultivars after large-scale testing and/or be involved in hybridization to develop varieties for drought-prone areas [32, 68].

### Construction of linkage map and mapping of QTLs governing drought tolerance

The linkage map is a prerequisite for the precise mapping of QTLs associated with drought tolerance [69]. In the present study, it was constructed using 5,165 GBS-based SNP markers widely distributed on 18 chromosomes. It covered a total length of 1671.87 cM with an average interval of 2.0 cM between marker loci (Table 2), indicating the availability of a large number of genome-wide SNP markers for the identification of genomic regions governing the traits under study. The GBS approach has earlier been successfully used for the construction of linkage maps [43, 70–72] and mapping of QTLs in *B. juncea* and its derived lines [29, 30, 73, 74].

A total of 29 QTLs were identified in the present study. Out of 29, 5 were responsible for WUE and drought tolerance, while the remaining 24 were responsible for additive effect explaining variation in various agro-morphological traits (Table 3 & 5). Results revealed that *B. carinata* has contributed about 17 QTLs (58.6% of total QTLs detected) which regulates 10 of the studied traits, suggesting that alleles from the *B. carinata* largely contributed to improving the drought tolerance in these ILs. DRMRIJ 31 contributed 12 (41.4%) of the 29 QTLs identified in different environments. This implies that positive loci governing agro-morphological traits under moisture deficit stress conditions were majorly contributed by *B. carinata* parent in the ILs.

Out of 17 QTLs identified in ILs, 10 (58.8% of total QTLs detected) were contributed by the B genome of *B. carinata*, while the rest seven (41.2%) were contributed by the A genome. It is likely that pairing and recombination between the B genomes of both species, as well as the higher frequency of recombination between the A and C genomes, resulted in the introgression of the genomic regions between *B.* *carinata* and *B. juncea* [75, 76]. The present study reports a larger number of QTLs for drought tolerance and improved WUE in the B genome. As also revealed in the previous study, a larger number of introgressed segments were observed in the B genome than in the A genome of the ILs [33]. This might be due to the conservation of novel alleles for drought tolerance in *B. carinata* [77], and their elimination in *B. juncea* during the course of evolution. It is expected as a consequence of nucleo-cytoplasmic interactions leading to considerable changes in the B genome of *B. juncea*, whereas it remains intact in *B. carinata* [78, 79].

In the present study, 23 major QTLs expressing high PV (> 10%) for agro-morphological traits, WUE and drought tolerance indices under moisture deficit stress conditions were identified (Table 3 & 5). A major QTL expressing PV upto 20.65% and flanked by markers *2B_65115276* and *2B_64577224* was identified to regulate the siliqua length (*qSL.2B.1*) trait in two different environments. A QTL governing seed per siliqua (*qSS.9A.1)*, flanked by markers *9A_38513927 and 9A_5962669*, explained PV upto 18.21% was detected in two different environments (Table 3). Both QTLs exhibited a positive additive effect, indicating that QTLs derived from the *B. carinata* parent (BC 4) are responsible for increasing siliqua length and seeds per siliqua in ILs under moisture deficit stress. Most of the QTLs reported to explain seed yield contributing traits showing positive additive effects, in the present study, were derived from *B. carinata* (BC 4), thus highlighting the usefulness of *B. carinata* in stabilizing productivity traits under drought stress conditions (Table 3). A few QTLs identified in current study for seed yield-contributing traits were found on the same chromosomes as in prior studies by Dhaka et al. [80], Rout et al., [72], and Aakanksha et al. [81]. Notably, these QTLs were associated with imparting drought tolerance and improving WUE under moisture deficit stress conditions in *B. carinata* derived introgression lines.

### QTL × environment interactions for morpho-physiological traits in ILs

The ability of genotypes to produce different phenotypes in a wide range of environments is mainly due to phenotypic plasticity arising from the interaction of QTLs with environments [82]. Therefore, understanding QTLs × environment interaction will help to select stable genotypes across environments, which further improves crop productivity [83, 84]. Previous studies also reported significant QTLs × environment interaction for yield-contributing traits in several crops, including mustard, and demonstrated the varied range of QTL expression with a change in environment [69, 80].

The present study also demonstrated the QTLs × environment interactions for morpho-physiological traits in *B. carinata* derived ILs under moisture deficit stress conditions (Table 4 & 6). Fifty QTLs were identified to govern agro-morphological traits (Table 4) and 16 QTLs for WUE/drought tolerance indices (Table 6) collectively demonstrated substantial QTL × environment interactions, indicating the existence of selectable genetic variations for phenotypic plasticity among these ILs. In addition, all the QTLs governing plant height, secondary branches per plant, main shoot length, seed yield/plant, oil content, days to 50% flowering and days to maturity, as well as a few QTLs for siliqua length, seeds/siliqua, biological yield/plant and 1000-seed weight were detected in specific environments, indicating their varied range of expression in different environments [69, 85]. The QTLs detected for siliqua length, seeds per siliqua, 1,000-seed weight and oil content observed small LOD (A x E) and high values for LOD (A), indicating their stable response to different environments (Table 4). This finding is consistent with previous studies of Singh et al. [86]; Singh et al. [87]; Singh et al. [88]; Binod et al. [89], which demonstrate the preponderance of additive gene action governing these traits.

The majority of QTLs for WUE and drought tolerance indices had low QTL × environment interactions (Table 6), revealing their stability across the environments. However, QTLs associated with WUE and MRP, namely *qWUE.7B.1*, *qWUE.7B.3*, *qWUE.7B.2* and *qMRP.8B.1*, had the highest QTL × environment interactions, implying differential expression pattern in response to different environments. The effect of environment on QTL expression demonstrates that QTL × environment interactions is a key component of genetic variation which can play an important role in defining future mustard breeding programs. The QTLs identified in the present study can be used in a wide range of environments or any specific environment based on their degree of QTL × environment interactions.

### Identification of co-localized QTLs and candidate genes in the QTL regions

The genomic regions containing multiple QTLs for different traits, also called QTL hotspots, enable simultaneous selection and accelerating the breeding progress through MAS [90]. The present study reported four co-localized QTLs, viz*.*, *qSS.7A.1*, *qSL.7A.1*, *qWUE.7A.2* and *qDTI.7A.1* on chromosome 7A (Table 7; Fig. 6). QTLs for siliqua length (*qSL.9A.1*) and seeds per siliqua (*qSS.9A.1*), on the other hand, were reported at the same position with the same set of flanking markers, indicating a pleiotropic effect. QTLs, viz., *qBY.3B.2*, *qMSL.3B.1*, *qSY.3B.1*, *qSL.3B.1*, *qDF.3B.1*, *qPH.3B.1*, *qDM.3B.1* and *qWUE.3B.1* were observed to be co-localized on chromosome 3B, revealing a QTL hotspot that can be further exploited through MAS for improving trait values under moisture deficit stress conditions (Table 7).

Seventeen candidate genes, known to regulate various pathways related to biotic and abiotic stress tolerance, were identified within QTL regions through in silico analysis in this study (Table 7). The genomic region of *qMSL.6A.1* contains *SOS2like* and *NPR1* genes associated with salt tolerance and salicylic acid-mediated systemic acquired resistance (SAR) pathways, respectively [50, 51]. QTLs *qSS.7A.1* and *qSL.7A.1* carry the *FAE1 KCS* gene involved in erucic acid production [91, 92]. Three candidate genes, *HOT5*, *DNAJA1*, and *NIA1*, were identified in the *qWUE.7A.2* and *qDTI.7A.1* QTL regions, known for their role in inducing abiotic stress tolerance [53–55]. The *qMRP.7A.1* QTL, associated with moisture deficit stress, harbours the *BRI1* gene linked to drought stress tolerance [56]. *RF21* and *ycf2* genes were co-localized in the *qSL.9A.1* and *qSS.9A.1* QTL regions, respectively while *WRKY-33* gene was found in the *qSB.3B.1* QTL, associated with abiotic stress responses [58]. *PAL* and *SAMS2* genes were detected in *qBY.3B.2* and *qMSL.3B.1* QTL regions, respectively, known for their involvement in drought and salt tolerance [59–62, 93, 94]. Lastly, the *SOS2like* gene involved in plant response to abiotic stresses found to be co-localized with *qSY.3B.2* and *qSL.3B.1* QTLs for seed yield and siliqua length respectively [50].

Similarly, the genomic region of co-localized QTLs *qSL.3B.2* and *qTSW.3B.1* encompasses the *MAPK3* gene, which is reported to impart drought tolerance in tomato [63]. The drought tolerance index QTL *qDTI.6B.1* contained four genes: *SOS2* [50]; *ERF* [64]; *BRI1* [56]; and *WRR1* [65]. Three of these four genes, viz., *SOS2*, *ERF* and *BRI1*, were reported to be involved in plant responses to abiotic stresses [50, 56, 64]. The *SUS* gene encoding sucrose synthase 09 [66] was identified within the genomic region of QTL *qWUE.7B.1*, and identified to be responsible for improving water use efficiency under moisture deficit stress conditions (Table 7). Thus, QTLs detected in multiple environments and carrying already known genes associated with drought tolerance can also be used to identify drought-responsive key candidate genes and their markers following the transcriptomic approach by mapping the transcripts over the reference sequence as reported in crops including Brassicas [95, 96]. Furthermore, the co-localized QTLs conferring drought tolerance and/or improved WUE can be further subjected to fine mapping and validation for their wider applications in mustard breeding through marker-assisted selection. The material and information generated from this study are expected to have long-term implications for the development of drought-tolerant mustard varieties.

## Conclusion

The study demonstrated the potential of *B. carinata-*derived *B. juncea* ILs for improving drought tolerance and WUE in *B. juncea* cultivar DRMRIJ 31 and provided insights into the genetic basis of yield-contributing traits under moisture deficit stress conditions. Significant differences for yield-contributing agro-morphological traits were observed among the ILs, evaluated under both rainfed and irrigated conditions, indicating the creation of sufficient genetic variability through interspecific hybridization. These ILs exhibited higher WUE and drought tolerance indices than their parents; moreover, some of these lines also showed higher phenotypic values under both rainfed and irrigated conditions. The genotyping by sequencing (GBS)-based linkage map helped identify the genomic regions associated with drought tolerance and improved WUE. Twenty-nine QTLs for seed yield-related traits were discovered, with a significant contribution from the B genome of *B. carinata*. Significant QTL-environment interactions were also observed, indicating the influence of the environment on the expression of the studied traits. QTL hotspots for various traits were identified in the present study, which will further provide opportunities for marker-assisted selection. In silico analysis identified 17 candidate genes involved in stress tolerance pathways within the QTL regions. The material and information generated from the present study have demonstrated the usefulness of interspecific hybridization among Brassicas in creating selectable novel genetic variability and paved the way for the development of high-yielding varieties with better WUE and improved productivity in water-scarce regions.

### Supplementary Information

**Additional file 1:** **Supplementary Table**. Seed yield, water use efficiency (WUE), drought tolerance index (DTI), and mean relative performance (MRP) of introgression lines (ILs) along with their parents evaluated under rainfed (RE) and irrigated (IE) conditions in three environments.

## Data Availability

All data generated or analyzed during this study are included in the paper or supplementary information.
