# Supplementary material for: Dissection of QTLs conferring drought tolerance in B. carinata derived B. juncea introgression lines
Source: BMC Plant Biol. 2023 Dec 21;23:664. doi: 10.1186/s12870-023-04614-z (PMC10740311; doi:10.1186/s12870-023-04614-z)
Supplement: Supplementary file 1 — Additional file 1: Supplementary Table. Seed yield, water use efficiency (WUE), drought tolerance index (DTI), and mean relative performance (MRP) of introgression lines (ILs) along with their parents evaluated under rainfed (RE) and irrigated (IE) conditions in three environments. [file 12870_2023_4614_MOESM1_ESM.docx]

Supplementary Table. Seed yield, water use efficiency (WUE), drought tolerance index (DTI), and mean relative performance (MRP) of introgression lines (ILs) along with their parents evaluated under rainfed (RE) and irrigated (IE) conditions in three environments

| Parents/ILs | Seed yield (kg/ha) | | WUE (kg/m^3^) | | DTI | MRP | Seed yield (kg/ha) | | WUE (kg/m^3^) | | DTI | MRP | Seed yield (kg/ha) | | WUE (kg/m^3^) | | DTI | MRP |
| --- | --- | --- | --- | --- | --- | --- | --- | --- | --- | --- | --- | --- | --- | --- | --- | --- | --- | --- |
|  | RE1 | IE1 | RE1 | IE1 |  |  | RE2 | IE2 | RE2 | IE2 |  |  | RE3 | IE3 | RE3 | IE3 |  |  |
| IL79 | 1865 | 2969 | 1.63 | 1.38 | 0.54 | 1.64 | 2273 | 2782 | 3.27 | 1.64 | 0.70 | 1.69 | 2200 | 2672 | 6.08 | 1.96 | 1.00 | 2.11 |
| IL80 | 1779 | 4483 | 1.55 | 2.09 | 0.77 | 2.08 | 2434 | 1848 | 3.50 | 1.09 | 0.50 | 1.43 | 1527 | 2141 | 4.22 | 1.57 | 0.56 | 1.59 |
| IL81 | 1484 | 2613 | 1.29 | 1.22 | 0.38 | 1.38 | 2333 | 1848 | 3.36 | 1.09 | 0.48 | 1.40 | 2165 | 3806 | 5.98 | 2.79 | 1.41 | 2.57 |
| IL82 | 2740 | 4969 | 2.39 | 2.31 | 1.32 | 2.60 | 1926 | 1396 | 2.77 | 0.82 | 0.30 | 1.11 | 1315 | 2354 | 3.63 | 1.73 | 0.53 | 1.58 |
| IL83 | 1095 | 1842 | 0.95 | 0.86 | 0.20 | 0.99 | 2832 | 2352 | 4.07 | 1.39 | 0.74 | 1.73 | 1563 | 3027 | 4.32 | 2.22 | 0.81 | 1.97 |
| IL84 | 1372 | 3668 | 1.20 | 1.71 | 0.49 | 1.67 | 2626 | 1332 | 3.78 | 0.79 | 0.39 | 1.32 | 996 | 2389 | 2.75 | 1.75 | 0.41 | 1.44 |
| IL85 | 1512 | 3798 | 1.32 | 1.77 | 0.56 | 1.76 | 2669 | 1943 | 3.84 | 1.15 | 0.58 | 1.54 | 1775 | 2425 | 4.90 | 1.78 | 0.74 | 1.82 |
| IL86 | 2065 | 2376 | 1.80 | 1.11 | 0.48 | 1.53 | 2866 | 2093 | 4.12 | 1.24 | 0.67 | 1.66 | 1846 | 1433 | 5.10 | 1.05 | 0.45 | 1.44 |
| IL87 | 2033 | 3863 | 1.77 | 1.80 | 0.76 | 1.98 | 2829 | 2729 | 4.07 | 1.61 | 0.86 | 1.86 | 1563 | 2460 | 4.32 | 1.81 | 0.66 | 1.73 |
| IL88 | 1923 | 3582 | 1.68 | 1.67 | 0.67 | 1.85 | 3246 | 2677 | 4.67 | 1.58 | 0.97 | 1.98 | 2094 | 1929 | 5.78 | 1.42 | 0.69 | 1.76 |
| IL89 | 3418 | 5880 | 2.98 | 2.74 | 1.95 | 3.14 | 3350 | 2993 | 4.82 | 1.77 | 1.12 | 2.12 | 2129 | 2070 | 5.88 | 1.52 | 0.75 | 1.83 |
| IL90 | 2457 | 4229 | 2.14 | 1.97 | 1.01 | 2.26 | 2902 | 2904 | 4.18 | 1.71 | 0.94 | 1.94 | 1209 | 3239 | 3.34 | 2.38 | 0.67 | 1.89 |
| IL91 | 3360 | 5580 | 2.93 | 2.60 | 1.82 | 3.03 | 2114 | 2490 | 3.04 | 1.47 | 0.59 | 1.54 | 2094 | 2247 | 5.78 | 1.65 | 0.80 | 1.89 |
| IL92 | 2037 | 4356 | 1.78 | 2.03 | 0.86 | 2.14 | 2753 | 1837 | 3.96 | 1.08 | 0.56 | 1.54 | 2661 | 2651 | 7.35 | 1.95 | 1.20 | 2.32 |
| IL93 | 1162 | 2400 | 1.01 | 1.12 | 0.27 | 1.19 | 2696 | 2238 | 3.88 | 1.32 | 0.67 | 1.65 | 2448 | 1893 | 6.76 | 1.39 | 0.79 | 1.91 |
| IL94 | 831 | 3666 | 0.72 | 1.71 | 0.30 | 1.46 | 2234 | 2621 | 3.21 | 1.55 | 0.65 | 1.62 | 1350 | 1008 | 3.73 | 0.74 | 0.23 | 1.04 |
| IL95 | 1564 | 3951 | 1.36 | 1.84 | 0.60 | 1.83 | 3042 | 2586 | 4.38 | 1.53 | 0.88 | 1.88 | 2165 | 1397 | 5.98 | 1.03 | 0.52 | 1.57 |
| IL96 | 2412 | 2523 | 2.10 | 1.18 | 0.59 | 1.71 | 2520 | 1770 | 3.63 | 1.04 | 0.50 | 1.43 | 1386 | 1752 | 3.83 | 1.29 | 0.41 | 1.36 |
| IL97 | 3349 | 4476 | 2.92 | 2.08 | 1.45 | 2.68 | 2680 | 2842 | 3.86 | 1.68 | 0.85 | 1.85 | 2832 | 1758 | 7.82 | 1.29 | 0.85 | 2.03 |
| IL98 | 2962 | 2683 | 2.58 | 1.25 | 0.77 | 1.97 | 2651 | 2663 | 3.81 | 1.57 | 0.79 | 1.78 | 2867 | 1616 | 7.92 | 1.19 | 0.79 | 1.98 |
| IL99 | 2091 | 3328 | 1.82 | 1.55 | 0.67 | 1.84 | 3344 | 3714 | 4.81 | 2.19 | 1.38 | 2.36 | 2194 | 1651 | 6.06 | 1.21 | 0.62 | 1.69 |
| IL100 | 2795 | 2861 | 2.44 | 1.33 | 0.77 | 1.96 | 2272 | 3035 | 3.27 | 1.79 | 0.77 | 1.77 | 2053 | 1439 | 5.67 | 1.06 | 0.50 | 1.54 |
| IL101 | 4513 | 5610 | 3.93 | 2.61 | 2.45 | 3.48 | 2548 | 3443 | 3.67 | 2.03 | 0.98 | 2.00 | 3257 | 2005 | 9.00 | 1.47 | 1.12 | 2.32 |
| IL102 | 3758 | 2780 | 3.28 | 1.29 | 1.01 | 2.31 | 2908 | 3107 | 4.18 | 1.83 | 1.01 | 2.01 | 2478 | 1651 | 6.84 | 1.21 | 0.70 | 1.82 |
| IL103 | 2593 | 3305 | 2.26 | 1.54 | 0.83 | 2.02 | 3561 | 3930 | 5.12 | 2.32 | 1.56 | 2.50 | 3221 | 2289 | 8.90 | 1.68 | 1.26 | 2.42 |
| IL104 | 4450 | 6338 | 3.88 | 2.95 | 2.73 | 3.68 | 3013 | 3487 | 4.33 | 2.06 | 1.17 | 2.17 | 3328 | 2926 | 9.19 | 2.15 | 1.66 | 2.74 |
| IL105 | 3255 | 5295 | 2.84 | 2.47 | 1.67 | 2.90 | 1875 | 3052 | 2.70 | 1.80 | 0.64 | 1.65 | 2230 | 2041 | 6.16 | 1.50 | 0.78 | 1.87 |
| IL106 | 3485 | 4903 | 3.04 | 2.28 | 1.66 | 2.86 | 2987 | 3513 | 4.30 | 2.07 | 1.17 | 2.17 | 3292 | 2749 | 9.09 | 2.02 | 1.55 | 2.65 |
| IL107 | 2569 | 2364 | 2.24 | 1.10 | 0.59 | 1.72 | 2030 | 2461 | 2.92 | 1.45 | 0.56 | 1.50 | 2726 | 2253 | 7.53 | 1.65 | 1.05 | 2.18 |
| IL108 | 3697 | 2561 | 3.22 | 1.19 | 0.92 | 2.22 | 2893 | 3402 | 4.16 | 2.01 | 1.10 | 2.10 | 2194 | 1828 | 6.06 | 1.34 | 0.69 | 1.76 |
| IL109 | 2453 | 2813 | 2.14 | 1.31 | 0.67 | 1.82 | 3226 | 2616 | 4.64 | 1.54 | 0.94 | 1.95 | 1663 | 2678 | 4.59 | 1.97 | 0.76 | 1.87 |
| IL110 | 3762 | 3483 | 3.28 | 1.62 | 1.27 | 2.53 | 2205 | 3019 | 3.17 | 1.78 | 0.74 | 1.75 | 1663 | 2218 | 4.59 | 1.63 | 0.63 | 1.68 |
| IL111 | 1015 | 1695 | 0.88 | 0.79 | 0.17 | 0.92 | 2497 | 2253 | 3.59 | 1.33 | 0.63 | 1.59 | 1982 | 2289 | 5.47 | 1.68 | 0.77 | 1.86 |
| IL112 | 4278 | 3765 | 3.73 | 1.75 | 1.56 | 2.82 | 2912 | 3029 | 4.19 | 1.79 | 0.98 | 1.99 | 3044 | 2678 | 8.41 | 1.97 | 1.39 | 2.50 |
| IL113 | 3615 | 3484 | 3.15 | 1.62 | 1.22 | 2.47 | 2597 | 2924 | 3.74 | 1.72 | 0.84 | 1.85 | 1592 | 2076 | 4.40 | 1.52 | 0.56 | 1.59 |
| IL114 | 4115 | 2501 | 3.59 | 1.16 | 1.00 | 2.36 | 2895 | 3449 | 4.17 | 2.03 | 1.11 | 2.12 | 2053 | 2572 | 5.67 | 1.89 | 0.90 | 2.01 |
| IL115 | 3250 | 4111 | 2.83 | 1.91 | 1.29 | 2.53 | 3242 | 3637 | 4.66 | 2.15 | 1.31 | 2.30 | 2265 | 2253 | 6.26 | 1.65 | 0.87 | 1.97 |
| IL116 | 4092 | 2293 | 3.57 | 1.07 | 0.91 | 2.29 | 3148 | 3145 | 4.53 | 1.86 | 1.10 | 2.10 | 2159 | 2218 | 5.96 | 1.63 | 0.82 | 1.91 |
| IL117 | 3545 | 3049 | 3.09 | 1.42 | 1.05 | 2.31 | 3504 | 4176 | 5.04 | 2.46 | 1.63 | 2.57 | 2336 | 2430 | 6.45 | 1.78 | 0.97 | 2.08 |
| IL118 | 2867 | 3068 | 2.50 | 1.43 | 0.85 | 2.06 | 3433 | 3730 | 4.94 | 2.20 | 1.42 | 2.39 | 2690 | 1970 | 7.43 | 1.45 | 0.91 | 2.05 |
| IL119 | 2938 | 4623 | 2.56 | 2.15 | 1.32 | 2.57 | 3169 | 3895 | 4.56 | 2.30 | 1.37 | 2.36 | 2442 | 3245 | 6.75 | 2.38 | 1.35 | 2.46 |
| IL120 | 2843 | 3468 | 2.48 | 1.62 | 0.96 | 2.17 | 2689 | 3160 | 3.87 | 1.86 | 0.95 | 1.95 | 1876 | 2218 | 5.18 | 1.63 | 0.71 | 1.78 |
| IL121 | 3626 | 4197 | 3.16 | 1.95 | 1.47 | 2.70 | 3048 | 3062 | 4.39 | 1.81 | 1.04 | 2.04 | 2726 | 2926 | 7.53 | 2.15 | 1.36 | 2.46 |
| IL122 | 2011 | 2661 | 1.75 | 1.24 | 0.52 | 1.60 | 2193 | 2425 | 3.16 | 1.43 | 0.59 | 1.54 | 2194 | 1722 | 6.06 | 1.26 | 0.65 | 1.72 |
| IL123 | 3260 | 2274 | 2.84 | 1.06 | 0.72 | 1.96 | 2546 | 3202 | 3.66 | 1.89 | 0.91 | 1.92 | 3328 | 2395 | 9.19 | 1.76 | 1.36 | 2.52 |
| IL124 | 2686 | 1311 | 2.34 | 0.61 | 0.34 | 1.44 | 2374 | 2782 | 3.42 | 1.64 | 0.73 | 1.72 | 2761 | 2183 | 7.63 | 1.60 | 1.03 | 2.17 |
| IL125 | 3408 | 2717 | 2.97 | 1.27 | 0.90 | 2.15 | 3326 | 3402 | 4.79 | 2.01 | 1.26 | 2.25 | 3824 | 2749 | 10.56 | 2.02 | 1.80 | 2.89 |
| IL126 | 3815 | 3364 | 3.33 | 1.57 | 1.24 | 2.51 | 3407 | 4121 | 4.90 | 2.43 | 1.56 | 2.52 | 3080 | 2289 | 8.51 | 1.68 | 1.20 | 2.36 |
| IL127 | 2858 | 3261 | 2.49 | 1.52 | 0.90 | 2.11 | 3407 | 3379 | 4.90 | 1.99 | 1.28 | 2.27 | 2124 | 2891 | 5.87 | 2.12 | 1.05 | 2.17 |
| IL128 | 3813 | 4008 | 3.32 | 1.87 | 1.48 | 2.71 | 3468 | 3379 | 4.99 | 1.99 | 1.30 | 2.29 | 3753 | 3316 | 10.37 | 2.43 | 2.12 | 3.09 |
| IL129 | 1732 | 2379 | 1.51 | 1.11 | 0.40 | 1.41 | 3111 | 3612 | 4.48 | 2.13 | 1.25 | 2.25 | 2832 | 2855 | 7.82 | 2.10 | 1.38 | 2.48 |
| IL130 | 2765 | 3159 | 2.41 | 1.47 | 0.85 | 2.05 | 3742 | 3965 | 5.38 | 2.34 | 1.65 | 2.58 | 2513 | 3847 | 6.94 | 2.82 | 1.65 | 2.74 |
| IL131 | 2948 | 3927 | 2.57 | 1.83 | 1.12 | 2.35 | 4038 | 4540 | 5.81 | 2.68 | 2.04 | 2.87 | 2938 | 3387 | 8.12 | 2.49 | 1.70 | 2.75 |
| IL132 | 1812 | 3039 | 1.58 | 1.42 | 0.53 | 1.64 | 2788 | 3170 | 4.01 | 1.87 | 0.98 | 1.99 | 1592 | 2183 | 4.40 | 1.60 | 0.59 | 1.63 |
| IL133 | 1904 | 3634 | 1.66 | 1.69 | 0.67 | 1.86 | 2954 | 3405 | 4.25 | 2.01 | 1.12 | 2.13 | 3044 | 2643 | 8.41 | 1.94 | 1.37 | 2.49 |
| IL134 | 2712 | 3044 | 2.36 | 1.42 | 0.80 | 1.99 | 4210 | 4709 | 6.06 | 2.78 | 2.21 | 2.98 | 2371 | 3103 | 6.55 | 2.28 | 1.26 | 2.37 |
| IL135 | 3144 | 3437 | 2.74 | 1.60 | 1.05 | 2.28 | 4305 | 4085 | 6.19 | 2.41 | 1.96 | 2.80 | 2726 | 3174 | 7.53 | 2.33 | 1.48 | 2.56 |
| IL136 | 2864 | 3876 | 2.50 | 1.81 | 1.08 | 2.31 | 3859 | 4020 | 5.55 | 2.37 | 1.73 | 2.63 | 2124 | 2678 | 5.87 | 1.97 | 0.97 | 2.08 |
| IL137 | 3570 | 3901 | 3.11 | 1.82 | 1.35 | 2.59 | 3214 | 3452 | 4.62 | 2.04 | 1.23 | 2.23 | 2088 | 2289 | 5.77 | 1.68 | 0.82 | 1.90 |
| IL138 | 2795 | 2147 | 2.44 | 1.00 | 0.58 | 1.74 | 2995 | 3630 | 4.31 | 2.14 | 1.21 | 2.21 | 2371 | 1616 | 6.55 | 1.19 | 0.65 | 1.76 |
| IL139 | 2509 | 3792 | 2.19 | 1.77 | 0.92 | 2.14 | 3913 | 4341 | 5.63 | 2.56 | 1.89 | 2.76 | 3257 | 3599 | 9.00 | 2.64 | 2.00 | 2.98 |
| IL140 | 3069 | 3535 | 2.68 | 1.65 | 1.05 | 2.28 | 3646 | 3463 | 5.25 | 2.04 | 1.40 | 2.38 | 2159 | 2360 | 5.96 | 1.73 | 0.87 | 1.97 |
| IL141 | 4039 | 3854 | 3.52 | 1.80 | 1.51 | 2.75 | 3453 | 3848 | 4.97 | 2.27 | 1.48 | 2.44 | 2584 | 837 | 7.14 | 0.61 | 0.37 | 1.53 |
| IL142 | 2537 | 3459 | 2.21 | 1.61 | 0.85 | 2.05 | 3879 | 3958 | 5.58 | 2.33 | 1.71 | 2.62 | 2478 | 3103 | 6.84 | 2.28 | 1.31 | 2.42 |
| IL143 | 1177 | 3500 | 1.03 | 1.63 | 0.40 | 1.54 | 2558 | 2475 | 3.68 | 1.46 | 0.70 | 1.68 | 1734 | 1439 | 4.79 | 1.06 | 0.43 | 1.39 |
| IL144 | 2348 | 3159 | 2.05 | 1.47 | 0.72 | 1.88 | 3793 | 4449 | 5.46 | 2.62 | 1.88 | 2.75 | 2159 | 2076 | 5.96 | 1.52 | 0.77 | 1.85 |
| IL145 | 1725 | 2873 | 1.50 | 1.34 | 0.48 | 1.56 | 2959 | 2365 | 4.26 | 1.40 | 0.78 | 1.78 | 1704 | 2118 | 4.71 | 1.55 | 0.62 | 1.66 |
| IL146 | 2389 | 3611 | 2.08 | 1.68 | 0.84 | 2.04 | 2273 | 2240 | 3.27 | 1.32 | 0.57 | 1.51 | 1704 | 1693 | 4.71 | 1.24 | 0.49 | 1.48 |
| IL147 | 3347 | 2644 | 2.92 | 1.23 | 0.86 | 2.11 | 3329 | 3369 | 4.79 | 1.99 | 1.25 | 2.24 | 2200 | 2011 | 6.08 | 1.48 | 0.76 | 1.84 |
| IL148 | 3892 | 2268 | 3.39 | 1.06 | 0.85 | 2.20 | 2936 | 3399 | 4.22 | 2.01 | 1.11 | 2.12 | 2554 | 1480 | 7.06 | 1.09 | 0.65 | 1.78 |
| IL149 | 3935 | 2559 | 3.43 | 1.19 | 0.98 | 2.31 | 3109 | 2763 | 4.47 | 1.63 | 0.96 | 1.96 | 1634 | 1303 | 4.51 | 0.96 | 0.36 | 1.29 |
| IL150 | 2444 | 1842 | 2.13 | 0.86 | 0.44 | 1.51 | 2574 | 2368 | 3.70 | 1.40 | 0.68 | 1.65 | 1386 | 1551 | 3.83 | 1.14 | 0.37 | 1.28 |
| IL151 | 2816 | 3092 | 2.46 | 1.44 | 0.84 | 2.04 | 2504 | 2170 | 3.60 | 1.28 | 0.60 | 1.56 | 1811 | 1834 | 5.00 | 1.35 | 0.57 | 1.59 |
| IL152 | 1964 | 2481 | 1.71 | 1.16 | 0.47 | 1.53 | 2629 | 2415 | 3.78 | 1.42 | 0.71 | 1.69 | 1704 | 2082 | 4.71 | 1.53 | 0.61 | 1.64 |
| IL153 | 3566 | 2132 | 3.11 | 0.99 | 0.74 | 2.03 | 2828 | 2566 | 4.07 | 1.51 | 0.81 | 1.80 | 1704 | 1728 | 4.71 | 1.27 | 0.50 | 1.50 |
| IL154 | 4696 | 2559 | 4.09 | 1.19 | 1.16 | 2.60 | 2931 | 3377 | 4.22 | 1.99 | 1.10 | 2.11 | 2094 | 2366 | 5.78 | 1.74 | 0.85 | 1.94 |
| IL155 | 3391 | 2068 | 2.96 | 0.96 | 0.68 | 1.95 | 4058 | 3898 | 5.84 | 2.30 | 1.76 | 2.66 | 2094 | 2188 | 5.78 | 1.61 | 0.78 | 1.87 |
| IL156 | 3656 | 3461 | 3.19 | 1.61 | 1.23 | 2.48 | 3236 | 2657 | 4.66 | 1.57 | 0.96 | 1.97 | 2094 | 2436 | 5.78 | 1.79 | 0.87 | 1.97 |
| IL157 | 2303 | 1875 | 2.01 | 0.87 | 0.42 | 1.47 | 2863 | 2676 | 4.12 | 1.58 | 0.85 | 1.85 | 1811 | 2366 | 5.00 | 1.74 | 0.73 | 1.81 |
| IL158 | 3184 | 3310 | 2.78 | 1.54 | 1.02 | 2.25 | 2507 | 3176 | 3.61 | 1.87 | 0.89 | 1.90 | 1032 | 1870 | 2.85 | 1.37 | 0.33 | 1.25 |
| IL159 | 2414 | 1267 | 2.10 | 0.59 | 0.30 | 1.32 | 2151 | 2351 | 3.09 | 1.39 | 0.56 | 1.50 | 1669 | 2543 | 4.61 | 1.87 | 0.72 | 1.82 |
| IL160 | 2040 | 1051 | 1.78 | 0.49 | 0.21 | 1.11 | 1270 | 1287 | 1.83 | 0.76 | 0.18 | 0.85 | 642 | 1622 | 1.77 | 1.19 | 0.18 | 0.96 |
| IL161 | 4226 | 2877 | 3.68 | 1.34 | 1.18 | 2.52 | 1501 | 2122 | 2.16 | 1.25 | 0.35 | 1.21 | 2590 | 2684 | 7.15 | 1.97 | 1.19 | 2.30 |
| IL162 | 2261 | 1547 | 1.97 | 0.72 | 0.34 | 1.35 | 1786 | 1770 | 2.57 | 1.04 | 0.35 | 1.19 | 1704 | 1445 | 4.71 | 1.06 | 0.42 | 1.38 |
| IL163 | 2670 | 2953 | 2.33 | 1.38 | 0.76 | 1.94 | 2615 | 2867 | 3.76 | 1.69 | 0.83 | 1.83 | 1421 | 2932 | 3.93 | 2.15 | 0.71 | 1.86 |
| IL164 | 2924 | 2093 | 2.55 | 0.97 | 0.59 | 1.77 | 2921 | 2330 | 4.20 | 1.37 | 0.76 | 1.76 | 1775 | 2295 | 4.90 | 1.68 | 0.70 | 1.76 |
| IL165 | 2296 | 2503 | 2.00 | 1.17 | 0.56 | 1.66 | 3280 | 2267 | 4.72 | 1.34 | 0.83 | 1.86 | 3086 | 1870 | 8.52 | 1.37 | 0.99 | 2.19 |
| Minimum | 831 | 1051 | 0.72 | 0.49 | 0.17 | 0.92 | 1270 | 1287 | 1.83 | 0.76 | 0.18 | 0.85 | 642 | 837 | 1.77 | 0.61 | 0.18 | 0.96 |
| Maximum | 4696 | 6338 | 4.09 | 2.95 | 2.73 | 3.68 | 4305 | 4709 | 6.19 | 2.78 | 2.21 | 2.98 | 3824 | 3847 | 10.56 | 2.82 | 2.12 | 3.09 |
| DRMRIJ 31 | 3309 | 4542 | 2.88 | 2.12 | 1.46 | 2.68 | 3635 | 3458 | 5.23 | 2.04 | 1.40 | 2.37 | 2426 | 3010 | 5.70 | 2.21 | 1.25 | 2.36 |
| BC 4 | 1406 | 895 | 1.23 | 0.42 | 0.12 | 0.82 | 978 | 907 | 1.41 | 0.54 | 0.10 | 0.63 | 1832 | 1126 | 5.06 | 0.83 | 0.35 | 1.31 |
